# A spatial genetics approach to inform vector control of tsetse flies (*Glossina fuscipes fuscipes*) in Northern Uganda

**DOI:** 10.1002/ece3.4050

**Published:** 2018-05-04

**Authors:** Norah Saarman, Mary Burak, Robert Opiro, Chaz Hyseni, Richard Echodu, Kirstin Dion, Elizabeth A. Opiyo, Augustine W. Dunn, Giuseppe Amatulli, Serap Aksoy, Adalgisa Caccone

**Affiliations:** ^1^ Department of Ecology and Evolutionary Biology Yale University New Haven Connecticut; ^2^ Department of Biology Faculty of Science Gulu University Gulu Laroo Division Uganda; ^3^ Department of Biology University of Mississippi Oxford Massachusetts; ^4^ Division of Genetics and Genomics Boston Children's Hospital Boston Massachusetts; ^5^ Department of GeoComputation and Spatial Science Yale School of Forestry and Environmental Studies New Haven Connecticut; ^6^ Department of Epidemiology of Microbial Diseases Yale School of Public Health New Haven Connecticut

**Keywords:** landscape genetics, maximum entropy model, sleeping sickness, spatial genetics, tsetse fly, vector control

## Abstract

Tsetse flies (genus *Glossina*) are the only vector for the parasitic trypanosomes responsible for sleeping sickness and nagana across sub‐Saharan Africa. In Uganda, the tsetse fly *Glossina fuscipes fuscipes* is responsible for transmission of the parasite in 90% of sleeping sickness cases, and co‐occurrence of both forms of human‐infective trypanosomes makes vector control a priority. We use population genetic data from 38 samples from northern Uganda in a novel methodological pipeline that integrates genetic data, remotely sensed environmental data, and hundreds of field‐survey observations. This methodological pipeline identifies isolated habitat by first identifying environmental parameters correlated with genetic differentiation, second, predicting spatial connectivity using field‐survey observations and the most predictive environmental parameter(s), and third, overlaying the connectivity surface onto a habitat suitability map. Results from this pipeline indicated that net photosynthesis was the strongest predictor of genetic differentiation in *G. f. fuscipe*s in northern Uganda. The resulting connectivity surface identified a large area of well‐connected habitat in northwestern Uganda, and twenty‐four isolated patches on the northeastern margin of the *G. f. fuscipes* distribution. We tested this novel methodological pipeline by completing an ad hoc sample and genetic screen of *G. f. fuscipes* samples from a model‐predicted isolated patch, and evaluated whether the ad hoc sample was in fact as genetically isolated as predicted. Results indicated that genetic isolation of the ad hoc sample was as genetically isolated as predicted, with differentiation well above estimates made in samples from within well‐connected habitat separated by similar geographic distances. This work has important practical implications for the control of tsetse and other disease vectors, because it provides a way to identify isolated populations where it will be safer and easier to implement vector control and that should be prioritized as study sites during the development and improvement of vector control methods.

## 1 INTRODUCTION

Tsetse flies (genus *Glossina*) are the only vectors of the trypanosome parasites that cause animal African trypanosomiasis (AAT) and human African trypanosomiasis (HAT), respectively, known as nagana and sleeping sickness. Together, the animal and human diseases pose health threats and economic burdens to vast regions of sub‐Saharan Africa where they are endemic (Budd, 1999; Committee, 1998; Diall et al., 2017; Murray & Lopez, 1996; PAAT, 2000; Simarro, Diarra, Ruiz Postigo, Franco, & Jannin, 2011; Simarro, Franco, Diarra, Postigo, & Jannin, 2012). The parasites responsible for these diseases form a group of closely related taxa within the genus *Trypanosoma* that requires the tsetse fly vectors to complete their life cycle, and for transmission between mammal hosts. The animal infective form of sleeping sickness, AAT, is caused by multiple *Trypanosoma* parasites in sub‐Saharan Africa*,* including *T. congolense, T. vivax,* and *T. brucei brucei*. The human‐infective form of sleeping sickness, HAT, is caused by parasites that are closely related to *T. b. brucei* (Balmer et al., 2010; Sistrom et al., 2014) with two distinct host‐evasion types that cause unique disease symptoms known as “chronic” and “acute” sleeping sickness. Although the parasites that cause these two forms of the human disease are currently known in the literature as subspecies *T. b. gambiense* and *T. b. rhodesiense,* respectively, the formal taxonomic rank is under revision (Berriman et al., 2005; Echodu et al., 2015; Gibson, Marshall, Marshall, & Godfrey, 1980; Jackson et al., 2010; Sistrom et al., 2016). Regardless of taxonomy, both forms of the disease cause serious human illness and are difficult to treat, and the specific drug treatment course depends on the type and stage of the infection (Fèvre, Picozzi, Jannin, Welburn, & Maudlin, 2006; Fèvre, Wissmann, Welburn, & Lutumba, 2008). Furthermore, for all forms of the disease including AAT, there are no vaccines available (Diall et al., 2017), and the drugs for treatment are expensive, can cause severe side effects, and are difficult to administer in remote villages where the disease is most prevalent (Simarro et al., 2011, 2012). Consequently, one of the most effective means of disease control is to reduce tsetse fly populations and thereby interrupt the transmission cycle.

Successful vector control relies on large‐scale coordination of on‐the‐ground measures that are based on detailed knowledge of the vector's distribution, movement patterns, and connectivity across a landscape. Without large‐scale coordination, control efforts often result in reemergence of the vector after the intervention program has been halted (Aksoy, Caccone, Galvani, & Okedi, 2013; Bouyer et al., 2015; Manangwa et al., 2015; Okeyo et al., 2017; Opiro et al., 2016, 2017). One strategy for coordination relies on using environmental data to model the vectors’ movement patterns, or connectivity, across the landscape. Connectivity is difficult to model accurately because it depends on many factors including the species physiological requirements, such as thermal tolerance and metabolic limits, and current and past evolutionary forces acting on the resident populations. Nonetheless, models of connectivity across landscapes have greatly improved with advances in the quality and resolution of publicly available environmental data, and with the development of complex computational tools. For example, field surveys that provide GPS locations of vector presence can be combined with high‐resolution satellite imagery to model landscape connectivity and to identify regions of priority in vector control planning (Elith et al., 2011; Wint & Rogers, 2000). Another advance combines genetic data with high‐resolution environmental data to model genetic differentiation across the landscape, thereby inferring areas of probable species movement (Dyer, 2015; Manel & Holderegger, 2013; Segelbacher et al., 2010). These, and gene flow‐related methods (Bouyer et al., 2015; McRae, Dickson, Keitt, & Shah, 2008; McRae, Shah, & Mohapatra, 2013), have greatly improved opportunities to enhance the lasting benefits of vector control campaigns and can be applied to a range of vectors and regions where both ecological and genetic data are available.

Northern Uganda is a region with a special need for vector control because it is the only region in sub‐Saharan Africa that harbors both forms of HAT (Echodu et al., 2013; Hutchinson, Fèvre, Carrington, & Welburn, 2003; Picozzi, Carrington, & Welburn, 2008; Picozzi, Fevre, et al., 2008; Welburn & Odit, 2002). The distribution of the two forms of the human‐infective parasite is currently separated by <100 km in a region north of Lake Kyoga and is predicted to overlap in the near future (Picozzi et al., 2005; Welburn & Odit, 2002). Merging of the two distributions would complicate treatment and diagnosis, and could lead to the emergence of unforeseen pathologies if there were to be recombination between the closely related trypanosome parasites (Brun, Blum, Chappuis, & Burri, 2010; Burri, 2010; Dumas & Bouteille, 2000; Hamilton, Adams, Malele, & Gibson, 2008; Legros et al., 2002).

In northern Uganda, the tsetse fly species *Glossina fuscipes fuscipes* is the main disease vector (Okoth, 1980, 1982; Rogers & Robinson, 2004; Lehane et al., 2016). Although other *Glossina* species, such as *G. morsitans submorsitans* and *G. pallidipes,* occur in Uganda, their habitat preference does not overlap with regions of frequent human and cattle use (Okoth, 1980, 1982). Moreover, where they do occur, they occur very rarely and at low densities in habitat remote from *G. f. fuscipes* habitat and human use, making them epidemiologically irrelevant in the geographic region analyzed in this article. Control strategies for *G. f. fuscipes* have included insecticides sprayed aerially or directly onto cattle, traps, and baited targets (Lindh, Torr, Vale, & Lehane, 2009; Shaw et al., 2013; Tirados et al., 2015; Torr, Hargrove, & Vale, 2005). However, control initiatives implemented to date have experienced some setbacks due to resurgence of tsetse in treated areas because of either residual populations that were not eliminated or immigration from neighboring untreated areas (Aksoy et al., 2013; Opiro et al., 2016; Vreysen, Seck, Sall, & Bouyer, 2013). This highlights the need for improvements in vector control and monitoring (PATTEC, 2001; Simarro et al., 2011). The identification of isolated patches can provide useful natural settings for novel control methods and the improvement of old ones, very much as island locations have been argued by many as ideal sites to test novel protocols prior to their use in the main range of a species.

**Figure 1:**
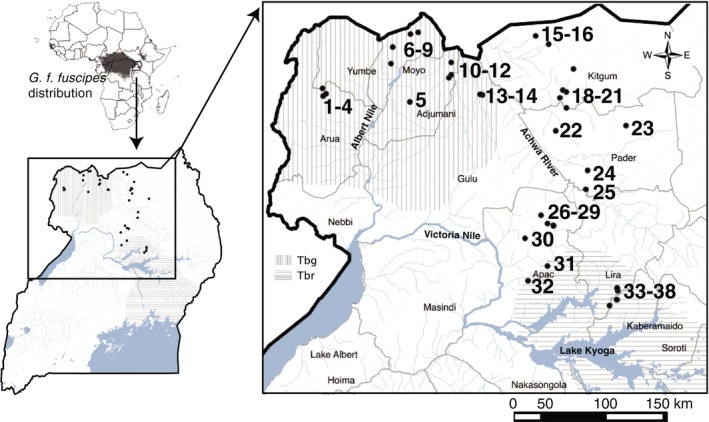
Map showing the spatial context of the study in northern Uganda. Sampling sites used for the population genetic input data are indicated as black dots. Numbers are the same as in Table S1 (Appendix S1), where information on these sites is reported. The map also shows the distribution of the two Trypanosoma parasites, *Trypanosoma brucei gambiense* to the west and *T. b. rhodesiense* to the east (gray lines), responsible for the chronic and acute form of the HAT disease. Water bodies (rivers and lakes) are shown in light gray with the major ones identified by name. The map also reports the district names for the region

**Figure 2:**
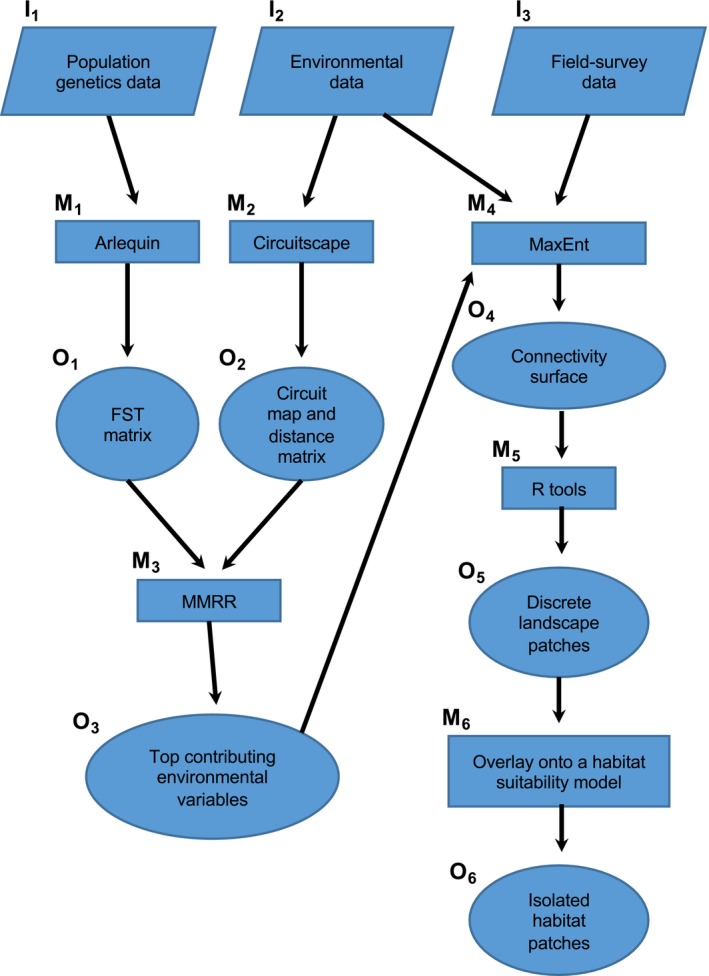
Flow diagram of the methodological pipeline. Inputs (I_1_, I_2,_ and I_3_) are shown as parallelograms, methods (M_1_–M_6_) as rectangles, and outputs (O_1_–O_6_) as ovals

In this study, our main goal was to identify isolated geographic regions for consideration in targeted vector control of *G. f. fuscipes* in northern Uganda (Figure 1) and to do so using a novel methodological pipeline that accounts for both evolutionary and ecological factors that can impact current levels of connectivity among tsetse populations. The main steps of the novel approach are summarized in Figure 2, including the types of inputs and outputs, and methods used. As input data, we used population genetic data (Figure 2: I_1_) from >800 *G. f. fuscipes* flies from 38 sampling sites (Figure 1) over a ~50,000 km^2^ region of northern Uganda (Opiro et al., 2017), environmental data (Figure 2: I_2_) and field‐survey data from > 300 locations (Figure 2: I_3_). This pipeline is novel because it integrates a landscape genetics approach (Figure 2: M_1–3_) with maximum entropy modeling using contemporary field‐survey observations (Figure 2: M_4_). The first step (Figure 2: M_1‐3_) identifies the major environmental parameters correlated with the accumulation of genetic differentiation (Figure 2: O_3_). In this first step, we anticipate detecting historical pathways of tsetse connectivity over hundreds to thousands of generations. The second step (Figure 2: M_4_) identifies connectivity between GPS coordinates currently inhabited with tsetse flies (Figure 2: O_4_). In this second step, we anticipate detecting contemporary pathways of tsetse connectivity over just several generations because predictions are dependent upon current observations of tsetse presence. The output is then analyzed (Figure 2: M_5‐6_) to identify habitat patches within ecologically suitable habitat with the least risk of long‐range recolonization over multiple generations (Figure 2: O_6_) and thereby allows for the identification of areas with the least risk of long‐range colonization over both short and long time scales ranging from several to thousands of generations. We validate this pipeline by (1) conducting a field‐survey searching for tsetse flies in geographic areas predicted by the model to have suitable habitat for *G. f. fuscipes* and to be isolated from other similarly suitable areas; (2) testing the level of genetic isolation of tsetse from one of these patches, as they should be more genetically differentiated for other tsetse populations from connected areas. We discuss how the integration of landscape genetics approaches with maximum entropy modeling provides potential advantages by accounting for historical and current movement patterns. We also discuss the possible immediate applications of this pipeline to help control tsetse flies in northern Uganda and consider its general applicability to identify isolated habitat in management and conservation of wild populations.

## 2 MATERIALS AND METHODS

### 2.1 Pipeline overview

The inputs for the pipeline include population genetic data, environmental data, and field‐survey presence data (Figure 2: I_1–3_). The population genetic data are used to estimate genetic differentiation in the software ARLEQUIN v.3.5 (Excoffier & Lischer, 2010; Figure 2: M_1_), which outputs a pairwise *F*_ST_ matrix among sampling sites (Figure 2: O_1_). The environmental data are used to create a resistance‐based landscape model in the program Circuitscape v.4.0.5 (McRae et al., 2008, 2013; Figure 2: M_2_), which outputs a circuit map for each environmental variable that is then used to estimate pairwise landscape resistance matrices among sampling sites (Figure 2: O_2_). We then used multiple matrix regression with randomization (MMRR; Figure 2: M_3_) in the R (R Development Core Team, 2011) package “PopGenReport” (Adamack and Gruber, 2014) to test for correlation between these two outputs (Figure 2: O_1_ and O_2_) for each environmental variable, while accounting for geographic distance and linear spatial autocorrelation. The environmental variable(s) identified as correlated with genetic differentiation (Figure 2: O_3_) are then combined with field‐survey presence data (Figure 2: I_3_) in a maximum entropy model using MaxEnt (Elith et al., 2011); Figure 2: M_4_) to build a connectivity surface (Figure 2: O_4_). This connectivity surface reflects the genetic differentiation among populations that exceeds isolation by distance (IBD; Wright, 1943). The connectivity surface (Figure 2: O_4_) is then used as input to a spatial clustering algorithm in the R package “SDMTools” (VanDerWal, Falconi, Januchowski, Shoo, & Storlie, 2014; Figure 2: M_5_) to identify discrete isolated landscape patches (Figure 2: O_5_). Finally, discrete isolated landscape patches (Figure 2: O_5_) are overlaid onto a habitat suitability model to yield predictions of where populations are relatively isolated within ecologically suitable habitat (Figure 2: O_6_).

### 2.2 Pipeline inputs

#### 2.2.1 Population genetic data

We included analysis from 16 microsatellite loci from *G. f. fuscipes* from northern Uganda (Figure 1) that represented a subset of the samples described by Opiro et al., 2017 (data available at https://doi.org/10.5061/dryad.20b01). We used a subset in order to exclude sampling sites with divergent genetic backgrounds that could introduce patterns of divergence unrelated to current environmental factors. Table 1 lists information for the subset of 38 localities and 805 individuals used in this study to develop the model. The table also includes allelic richness (AR), observed heterozygosity (*H*_O_), expected heterozygosity (*H*_E_), the individual fixation index relative to the sample (*F*_IS_) as estimated using GENALEX v6.501 (Peakall & Smouse, 2006).

**Table 1:** Sampling locality details include sample number (#) from Figure 1, village, district, sample size (N), latitude (lat), longitude (long), and basic diversity statistics reported in Opiro et al. (2017)

No	Village	District	*N*	Lat	Long	AR	*H*_O_	*H*_E_	*F*_IS_	Ne
1	Duku	Arua	25	3.267	31.135	5.81	0.57	0.65	0.12	No estimate
2	Aina	Arua	19	3.304	31.119	5.94	0.67	0.68	0.01	No estimate
3	Gangu	Arua	20	3.252	31.123	5.56	0.59	0.65	0.09	179.5
4	Omugo	Arua	15	3.268	31.143	5.31	0.69	0.66	−0.06	No estimate
5	Osugo	Moyo	20	3.211	31.725	5.38	0.54	0.63	0.13	332.1
6	Belameling	Moyo	10	3.479	31.594	4.63	0.56	0.60	0.07	No estimate
7	Lea	Moyo	8	3.592	31.606	4.25	0.54	0.56	0.04	No estimate
8	Orubakulemi	Moyo	20	3.692	31.780	5.31	0.53	0.60	0.12	1358
9	Moyo	Adjumani	15	3.683	31.727	5.25	0.59	0.62	0.02	250
10	Olobo	Adjumani	24	3.402	32.011	5.06	0.59	0.61	0.04	124
11	Oringya	Adjumani	9	3.486	32.010	4.56	0.65	0.61	−0.08	66
12	Pagirinya	Adjumani	20	3.378	31.994	5.19	0.56	0.63	0.12	No estimate
13	Okidi	Amuru	26	3.260	32.224	6.13	0.55	0.62	0.10	216
14	Gorodona	Amuru	25	3.266	32.208	6.06	0.59	0.60	0.02	1245*
15	Ngomoromo	Lamwo	25	3.669	32.591	5.31	0.60	0.64	0.07	117
16	Pawor	Lamwo	13	3.612	32.682	4.63	0.56	0.61	0.09	17
17	Lagwel	Lamwo	17	3.441	32.853	4.50	0.46	0.56	0.15	899
18	Bola	Kitgum	25	3.293	32.782	5.50	0.57	0.62	0.07	No estimate
19	Tumangu	Kitgum	20	3.242	32.761	4.94	0.54	0.57	0.03	No estimate
20	Kitgum	Kitgum	20	3.282	32.805	5.06	0.59	0.62	0.04	No estimate
21	Kitgum	Kitgum	17	3.171	32.805	4.81	0.55	0.60	0.09	1171
22	Omido	Pader	15	3.011	32.732	5.13	0.56	0.59	0.07	No estimate
23	Pader	Pader	13	3.050	33.217	5.38	0.63	0.64	0.01	51
24	Kilak	Pader	21	2.740	32.950	5.56	0.59	0.63	0.05	673
25	Chua	Pader	25	2.607	32.938	5.50	0.59	0.58	−0.02	368
26	Ocala	Oyam	20	2.427	32.629	5.13	0.61	0.61	−0.03	131*
27	Akayo‐debe	Oyam	26	2.372	32.676	5.44	0.57	0.59	0.02	332
28	Koome	Oyam	15	2.360	32.715	5.06	0.54	0.61	0.12	253
29	Olepo	Kole	24	2.356	32.716	5.25	0.56	0.57	0.02	165
30	Acanikoma	Kole	25	2.270	32.521	5.88	0.55	0.59	0.07	248
31	Aputu‐Lwaa	Apac	29	2.079	32.676	5.13	0.56	0.58	0.02	No estimate
32	Apac	Apac	15	1.976	32.539	4.56	0.51	0.57	0.09	No estimate
33	Kaberamaido	Dokolo	64	1.908	33.160	5.13	0.54	0.56	0.05	1686
34	Aminakwach	Dokolo	30	1.924	33.156	4.69	0.52	0.54	0.05	197
35	Aminakwach	Dokolo	25	1.925	33.156	4.19	0.53	0.54	−0.02	1549* ^**§**^
36	Oculoi	Kaber‐amaido	20	1.847	33.154	4.44	0.58	0.54	−0.06	101*
37	Oculoi	Kaber‐amaido	25	1.847	33.153	4.69	0.57	0.56	−0.03	112
38	Kangai	Kaber‐amaido	20	1.803	33.103	4.44	0.49	0.54	0.11	208

Allelic richness (AR), observed heterozygosity (*H*_O_), expected heterozygosity (*H*_E_), and the individual fixation index relative to the sample (*F*_IS_) as estimated using GENALEX v6.501 (Peakall & Smouse, 2006), and Ne estimated with the LD method in NEESTIMATOR v2.01 (Do et al., 2013), “no estimate” indicates indistinguishable from infinite. The Ne estimate is marked if there was significant evidence (*p* value <.05) of a bottleneck under the TPM model (*), or with the mode‐shift test (§) implemented in BOTTLENECK (Piry et al., 1999).

To assess the levels and patterns of genetic differentiation among the 38 sampling sites targeted in this study, we used principal components analysis (PCA) and discriminant analysis of principal components (DAPC) with prior groupings based on sampling site and with the cross‐validation formula for optimizing number of principal components to retain, using the adegenet R library (Jombart et al., 2010, 2012). We also performed clustering analysis with STRUCTURE v2.3.4 (Pritchard & Stephens, 2000; Falush et al., 2003). Finally, we test for isolation by distance (IBD) with a Mantel test with 10,000 randomizations implemented in the adegenet R library (Jombart et al., 2008, 2011). For this test, genetic distances were generated using Reynolds, Weir, and Cockerham (1983) method and geographic distances, in km, were generated using the Java‐based “geographic matrix generator” v1.2.3 (Erst, 2017 downloaded November 2017).

To discuss the impact of demographic history on our predictions, we also present the results from Opiro et al. (2017) for these 38 sampling sites on effective population size (Ne) estimates obtained using the linkage disequilibrium (LD) method in NEESTIMATOR v2.01 (Do, Waples, Peel, Macbeth, & Tillet, 2013; Table 1), tests for population bottlenecks occurrence obtained under the TPM model and using the mode‐shift test in the software BOTTLENECK (Piry, Luikart, & Cornuet, 1999).

#### 2.2.2 Environmental data

We selected continuous environmental variables of possible relevance to *G. f. fuscipes* population genetic structure based on published *G. f. fuscipes* laboratory and field experiments, known physiological requirements of this species, and population genetics. Although this step has an unavoidable subjectivity, we minimize this using all the environmental variables that may be relevant to tsetse distribution and dispersal, given *G. f. fuscipes’* know ecological requirements. Habitat preference for humid, cooler environments (Cecchi, Mattiolo, Slingenbergh, & De La Rocque, 2008; Dyer et al., 2011; Hargrove et al., 1992; Langridge, Kernaghan, & Glover, 1963; Leak, 1999), and laboratory and field experiments that indicate a negative behavioral response of this species to high temperature and low humidity (Dyer et al., 2011; Hargrove & Brady, 1992; Pollock, 1982) lead us to include surface temperature and rainfall. Field data from mark–recapture and population genetics studies that indicate *G. f. fuscipes* disperses a maximum of 2.5–14 km, depended on vegetation types (Bouyer et al., 2007; Challier, 1982; Cuisance, February, Dejardin, & Filledier, 1985; Hyseni et al., 2012; Rogers, 1977) led us to include vegetation parameters.

We used 13 types of environmental data (Table S1, Appendix S1) from moderate‐resolution imaging spectroradiometer (MODIS) remote‐sensed satellite data. We choose data relevant to our target environmental variables from the NOAA, the Numerical Terradynamic Simulation Group, USGS, the Global Land Cover Facility (DiMiceli et al., 2011), and the Consultative Group on International Agricultural Research Consortium for Spatial Information. Water availability was measured in mean annual rainfall (RNF) and latent evaporation (LE), and also approximated with elevation (ELEV) measured in meters above mean sea level. Temperature was measured in mean annual daytime surface temperatures (DST) and nighttime surface temperatures (NST). Vegetative indices included the normalized difference vegetation index (NDVI), enhanced vegetation index (EVI), leaf area index (LAI), and mean annual tree cover (TC), and also approximated with photosynthesis indices including net photosynthesis (PSN), gross primary production (GPP), the fraction of photosynthetically active radiation (fPAR) and evapotranspiration (ET). All environmental variables were scaled to 1 km^2^ resolution and trimmed to the extent of our genetic sample. Thus, our starting environmental dataset consisted of 13 continuous environmental variables (Table S1, Appendix S1) at 1 km^2^ resolution for northern Uganda.

To exclude covarying variables, we estimated pairwise straight‐path distances between each of the 38 sampling sites for each of the 13 environmental variables and calculated the mean value encountered along these straight‐path distances, using the Zonal Statistics tool in ArcMap 10.3 (ESRI 2014). We then performed linear regressions between straight‐path means for each pair of variables, using the “pairs” function in R. We also carried out principal components analysis (PCA) in JMP v11.0 (SAS Institute Inc., Cary, NC, USA, 1989– 2007) to identify among the covarying variables the ones that explained the most variance. We used the results from these two types of analyses to select just one variable representative of any two or more variables that had a strong linear relationship (|*R*| > .80 and *p*‐value <.02) and that also explained the most variance in the PCA. This reduced the number of environmental variables used as input data from 13 to five: RNF, DST, NST, NDVI, and PSN (Figure 2: I_2_).

#### 2.2.3 Field‐survey data

We used data from Opiro et al. (2017) collected from 317 traps deployed in northern Uganda (latitude 1.542°–3.692° and longitude 31.119°–33.217°), using a field protocol previously published (Beadell et al., 2010; Echodu et al., 2013; Opiro et al., 2016, 2017). In brief, traps were set out in groups of 10‐15 that were placed at ~100‐m intervals, and each cluster was spaced > 5 km from neighboring sites to cover a large geographic area of ~46,500 km^2^. Coordinates from multiple traps (within <2 km distance) were averaged to provide a single geospatial coordinate per sample. Very scant information on *G. f. fuscipes* distribution was available for this region before these surveys, because of the area was difficult to access due to civil unrest that plagued this region from the 1980s through 2009. Table S2 (Appendix S1) summarizes the geographic information for the presence data, which are the inputs together with the environmental variable(s) correlated with genetic differentiation for the MaxEnt analysis to obtain a connectivity surface (Figure 2: I_3_, M_4_ and O_4_).

### 2.3 Pipeline methods

#### 2.3.1 M_1_: Genetic differentiation

We used pairwise *F*_ST_ estimates (Table S3, Appendix S1) from Opiro et al. (2017; data available on DRYAD https://doi.org/10.5061/dryad.20b01) to quantify levels of genetic differentiation among the 38 sites (Figure 1; Table S1, Appendix S1). This matrix together with a matrix of geospatial coordinates for each site made up the input data for the Circuitscape analysis (Figure 2: I_1–2_).

#### 2.3.2 M_2_: Circuit models of environmental connectivity

We used Circuitscape (McRae et al., 2013) to build models of environmental connectivity between the 38 sampling sites, using the five non covarying environmental variables selected (Figure 2: Ib). Circuitscape employs circuit theory to predict how environmental resistance impacts species connectivity across a landscape (e.g., it produces “current” maps). Each of the 38 sampling sites was considered an “electrical source” to each of the remaining sites acting as the “ground.” Environmental resistance between pairwise source‐to‐ground sites is measured by applying random‐walk theory on a surface that reflects the putative cost to tsetse fly movement based on the ecology of the species (McRae et al., 2013). We first created “geographic distance only” current map and pairwise resistance matrix to provide a control for IBD for the next method in the pipeline (Figure 2: M_3_). Then, to prepare the environmental data input as resistance surfaces, we followed an approach similar to that which is described in Wang, Savage, and Bradley Shaffer (2009). This involved assigning hypothetical costs (“resistance costs”) to tsetse movement to environmental or topographic features in the landscape using 11 bins ranging from (1) 1–100 scaled linearly in both the positive and negative directions, and (2) from 1–500 scaled exponentially in both the positive and negative directions. This created a total of 20 environment‐specific resistance surfaces (4 × 5 = 20) that were used as inputs for Circuitscape (Figure 2: M_2_; Table 2). We used this wide range of options to assign cost values as suggested by Wang et al. (2009) to reduce subjectivity of the analysis. The output was 20 current maps, which show the areas of predicted connectivity in the landscape, and their corresponding 20 resistance matrices (Figure 2: O_2_), calculated as the mean resistance value encountered along a straight path between each pair of sampling sites. We created resistance matrices from the current maps, so that we had one geographic‐distance‐only resistance matrix and 20 environment‐specific resistance matrices (Figure 2: O_2_) of the same dimensions as the *F*_ST_ matrix (Figure 2: O_2_) to use as input for the test for correlation of the two matrices (Figure 2: M_3_).

**Table 2:** Results from MMRR (Wang, 2013) and the partial Mantel tests (Manel et al., 2003) for correlation between least resistance distances and genetic differentiation (*F*_ST_), showing type of environmental variable modeled (type), a description of the variable (description), the modeled effect of the variable on connectivity (effect), the method of assigning resistance costs (cost method), the range and units used (range), and the *p*‐value of correlation

Type	Description	Effect	Cost method	Range	MMRR *p* value	Partial Mantel *p* value
Water availability	Mean annual rainfall (RNF)	+	1–100 (linear)	9.64–43.59 mm	.378	.187
		−	1–100 (linear)	9.64–43.59 mm	.202	.908
		+	1–500 (exponential)	9.64–43.59 mm	.312	.150
		−	1–500 (exponential)	9.64–43.59 mm	.154	.933
Temperature	Mean annual daytime surface temperature (DST)	+	1–100 (linear)	200.62–316.53°K	.305	.841
		−	1–100 (linear)	200.62–316.53°K	.275	.859
		+	1–500 (exponential)	200.62–316.53°K	.332	.840
		−	1–500 (exponential)	200.62–316.53°K	.261	.852
Temperature	Mean annual nighttime surface temperature (NST)	+	1–100 (linear)	174.67–296.13°K	.849	.559
		−	1–100 (linear)	174.67–296.13°K	.342	.825
		+	1–500 (exponential)	174.67–296.13°K	.759	.370
		−	1–500 (exponential)	174.67–296.13°K	.350	.829
Vegetative	Normalized difference vegetation index (NDVI)	+	1–100 (linear)	0.12–0.86 NDVI	.789	.605
		−	1–100 (linear)	0.12–0.86 NDVI	.252	.876
		+	1–500 (exponential)	0.12–0.86 NDVI	.834	.585
		−	1–500 (exponential)	0.12–0.86 NDVI	.252	.883
Photosynthesis	Net photosynthesis (PSN)	+	1–100 (linear)	−1.29 to 6.59 GPP‐MR	.244	.886
		−	1–100 (linear)	−1.29 to 6.59 GPP–MR	.073	.040*
		+	1–500 (exponential)	−1.29 to 6.59 GPP–MR	.141	.933
		−	1–500 (exponential)	−1.29 to 6.59 GPP–MR	.050*	.021*

For the cost methods, “1–100 (linear)” indicates 11 evenly spaced resistance costs bins (1, 10, 20, 30, 40, 50, 60, 70, 80, 90, 100), and “1–50 (exponential)” indicates 11 evenly spaced resistance costs bins (1, 25, 50, 75, 100, 150, 200, 250, 325, 400, and 500). The resistance surfaces with *p* values ≤.05 are marked with *.

**Table 3:** Contributions of each of the five independent environmental variables to the MaxEnt habitat suitability model (Elith et al., 2011) used to update the existing FAO's habitat suitability model (Wint & Rogers, 2000)

Environmental variable included	Other highly correlated variables not included	Contribution (%)
Mean annual rainfall (RNF)	None	46.8
Mean annual daytime surface temperature (DST)	None	10.2
Mean annual nighttime surface temperature (NST)	None	0.0
Normalized difference vegetation index (NDVI)	fPAR, LAI, TC, EVI, LE, ET	24.4
Net photosynthesis (PSN)	GPP, ELEV	18.6

We list the environmental variable input into the model, other highly correlated variables that were not included (Table S1, Appendix S1; Figures S5 and S6, Appendix S2), and the contribution of the variable to the final model update.

#### 2.3.3 M_3_: Testing for correlation with genetic differentiation

To quantify the contribution of the environment to genetic differentiation, we used a generalized matrix regression called multiple matrix regression with randomization (MMRR; Figure 2: M_3_; Wang, 2013). Other methods are available for assessing environmental association (reviewed by Hall & Beissinger, 2014), including linear mixed effect modeling, geographically weighted regression (Fotheringham, Brunsdon, & Charlton, 2002), generalized dissimilarity modeling (Ferrier, Manion, Elith, & Richardson, 2007; Thomassen et al., 2010), Bayesian geographic analysis (Gautier, 2015) and ordination methods such as redundancy analysis. However, these models did not provide clear advantages over MMRR and can have higher rates of type I errors (Kierepka & Latch, 2015). Given that there were no clear advantages of other methods, we chose to use MMRR for this study.

The input for the MMRR was the *F*_ST_ matrix (Figure 2: O_1_), the geographic‐distance‐only resistance matrix and the 20 environment‐specific resistance matrices (Figure 2: O_2_). Prior to using MMRR, we used R to test for violation of the two main assumptions of linear regression, normality of residuals and homogeneity of variance. The MMRR was performed using the Wang (2013) method implemented in the *lgrMMRR* function of the “PopGenReport” package (Adamack and Gruber 2014). We control for spatial autocorrelation by first performing a regression of the *F*_ST_ matrix on the geographic distance matrix and then using the residuals to estimate the effect of each of the 20 Circuitscape‐derived resistance matrices. This two‐step process allowed us to identify environmental variables correlated with genetic differentiation that exceeded the level expected under an IBD model.

We additionally provide results from partial Mantel tests (Manel, Schwartz, Luikart, & Taberlet, 2003) as a comparative and confirmatory tool. Mantel tests remain common in landscape genetics (Manel et al., 2003) and can perform better than other methods when the assumption of linearity is not violated (Kierepka & Latch, 2015; Shirk, Landguth, & Cushman, 2017; Zeller et al., 2016). However, use of Mantel tests is controversial because of weakness in accounting for spatial autocorrelation (Legendre & Fortin, 2010; Manel et al., 2003), so we interpret results of the partial Mantel test with caution and as a supplement to the results of the MMRR. The partial Mantel test was implemented with the “vegan” package (Oksanen et al., 2013) in R.

For both MMRR and the partial Mantel tests, we used 10,000 permutations to calculate empirically based *p*‐values, and only considered models that were significantly correlated with genetic differentiation (*p*‐value <.05; Figure 2: O_3_) as candidates of high influence on genetic differentiation. These models were selected as inputs for modeling a *G. f. fuscipes* connectivity surface (see below).

#### 2.3.4 M_4_: Connectivity surface

We used a maximum entropy model (MaxEnt; Figure 2: M_4a_) to produce a connectivity surface, using the environmental variables significantly correlated with genetic differentiation from the previous step (Figure 2: O_3_) and field‐survey presence data from 317 traps from northern Uganda (Figure 2: I_3_). MaxEnt was chosen because of its widespread use in modeling movement corridors (Liu, McShea, & Li, 2017; Poor, Loucks, Jakes, & Urban, 2012), because it provides an easy‐to‐use GUI interface, and because it is permanently open‐source through the American Museum of Natural History (Elith et al., 2011; Phillips, Anderson, Dudík, Schapire, & Blair, 2017). This step produced a connectivity surface (Figure 2: O_4a_) for *G. fuscipes* tsetse flies in northern Uganda that accounts for genetics of the target species in a specific region of interest. We used the default program parameters, which included a logistic output that provides a 0‐1 scale probability of a presence. We gave a 0% predicted probability of presence to large continuous water bodies, such as Lake Albert, Lake Kyoga, Victoria Nile, and Albert Nile (Figure 1), as studies have shown that stretches of >10 km of open water are effective barrier to *G. f. fuscipes* dispersal and gene flow (Beadell et al., 2010; Echodu et al., 2013). Note that in this step, we used MaxEnt in a univariate rather that multivariate analyses, as we only had one environmental variable of significant correlation with genetic differentiation (see results). Thus, the output of this analysis is not meant to be a habitat suitability model, but a connectivity surface reflecting environmentally driven genetic differentiation.

#### 2.3.5 M_5_: Identifying discrete landscape patches

To identify geographic regions with low connectivity to the main tsetse habitat (isolated landscape patches), we used the connectivity surface (Figure 2: O_4a_) as the input of a clustering analysis (Figure 2: M_5_). For the clustering analysis, we used the R packages “raster” (Hijmans, 2016), “rgdal” (Bivand, Keitt, & Rowlingson, 2017) and “dismo” (Hijmans, Phillips, Leathwick, & Elith, 2017) to find discrete patches within our connectivity surface (Appendix S3). We first converted the connectivity surface into a matrix in which pixels with scores >0.50 were selected as the “environment of interest” (value of 1), while the remaining pixels, including water bodies, were considered “background” (value of 0). To evaluate sensitivity of our analysis to cutoff decisions, we explored different cutoff scores from 0.35 to 0.65 and a minimum number of pixels of 2–7 (each pixel measuring 1 km^2^; Appendix S4). After finding that discrete patches were stable across many of the chosen cutoffs, we decided to use a cutoff of a score >0.50 with a minimum size of 4 pixels, because it produced a representative set of discrete patches useful in a management context (i.e., discrete but still large enough to be useful for testing vector control and monitoring strategies; Appendix S4). We then clustered together these discrete patches according to their distance from other patches and the known dispersal ability of tsetse flies (5–8 km; Challier, 1982; Cuisance et al., 1985; Bouyer et al., 2007) and chose to conservatively use the smallest value (5 km), as the upper limit to group together geographically close patches (Appendix S4). Thus, the final criteria to identify discrete landscape patches (Figure 2: O_5_) in northern Uganda were as follows: a final cutoff score of >0.5, a minimum size of 4 pixels, and a clustering range of 5 km.

#### 2.3.6 M_6_: Identifying the isolated habitat patches that fall within suitable habitat

We overlaid the resulting discrete landscape patches (Figure 2: O_5_) onto a habitat suitability model to identify and exclude the largest continuous patch of suitable habitat (referred to as the “main habitat belt” from here forward) for consideration as an isolated patch. For a habitat suitability model, we started with the existing FAO model (Wint & Rogers, 2000). However, after discovering that some of the observed GPS points of *G. f. fuscipes* field‐survey observations we had from our survey data fell outside of the FAO model, we decided to update the habitat suitability model using MaxEnt (Elith et al., 2011). To build this updated model, we used the same five environmental parameters and presence data prepared for the main analysis pipeline (Figure 2: I_1_ and I_2_), with the same default program parameters used to build the connectivity surface. We then merged this MaxEnt output with the existing FAO model (Wint & Rogers, 2000) using the maximum suitability of each pixel from the new MaxEnt and the existing FAO models as our final updated habitat suitability model. We used the maximum value for each pixel to make our prediction of suitable habitat more inclusive. If, through this method, we potentially over predicted suitability, we would also be under predicting areas of low suitability and the number of isolated patches, making this a conservative approach.

We overlaid the patches identified in M_5_ onto this updated habitat suitability map to identify patches that fell within areas modeled at above 25% suitability. In doing so, we had to exclude discrete patches located <5 km from the edge of the model because the clustering algorithm used could not account for connection with habitat outside of the study area, for example to the west where the main suitable habitat extends past the geographic frame considered.

### 2.4 Validation of the methodological pipeline using field and genetic data

To determine whether flies from isolated but suitable areas identified in our pipeline were (1) present and (2) more genetically isolated from neighboring areas than would be expected based on geographic distance alone, we conducted a field survey and collected samples along three transects that crossed five of the model‐defined isolated patches along the northern shore of Lake Kyoga. The survey was carried out over a period of 3 days for each transect in November of 2016. Presence data were collected by deploying 317 biconical tsetse traps as described in previous work (Beadell et al., 2010; Echodu et al., 2013; Opiro et al., 2016, 2017). Traps were set out in 10–15 per group and placed at ~100‐m intervals within each group. The GPS coordinates for points of suitable habitats falling in each of these transects were used to deploy on average 26 traps/transect (total of 106 traps) within ≤5 km of each transect. To maximize finding flies preference was given to sites close to water bodies, with suitable vegetation nearby. Site accessibility by road was also taken into consideration to increase survey efficiency. Flies were stored individually in 95% ethanol, and information on sex, collection date, trap number, and geographic coordinates of each trap was recorded.

One site was selected for genotypic analyses to test whether flies from this area were indeed more genetically isolated than flies from sampling sites in connected habitat separated by similar geographic distance, as this would lend support to our approach to identify isolated patch with tsetse flies using a combination of environmental, genetic, and field‐survey presence data. DNA was extracted from two to four legs using Qiagen DNeasy Blood and Tissue Kit (Qiagen, Germany), following manufacturer's protocols and stored at −20°C. Genotypic data from 16 microsatellite loci were collected using published protocols to be able to merge the new data with the existing genotypic database for this region (Opiro et al., 2017).

Pairwise *F*_ST_ between all samples from (Opiro et al., 2017) and the ad hoc sample were obtained using ARLEQUIN (Excoffier & Lischer, 2010) with Wright's statistics (Wright, 1951), following the variance method developed by (Weir & Cockerham, 1984), using 10,000 permutations to obtain exact *p*‐values. *F*_ST_ was adjusted for finite populations (Rousset, 1997), using the equation *F*_ST_/(1 − *F*_ST_), and then we compared the level of differentiation of the isolated patch to the closest sites that we sampled from within the main habitat (25–100 km away), to the level of differentiation found between pairs of sites within the main habitat belt that were separated by the same range of geographic distances (25–100 km), and tested the prediction that the ad hoc isolated patch would have higher differentiation than pairs from within the main habitat belt with a t‐test in JMP.

## 3 RESULTS

### 3.1 Population genetic data

The results of the PCA, DAPC, and STRUCTURE analyses are presented in Figures S1, S2, and S3, respectively (Appendix S2). The PCA and DAPC indicate that the majority of the variance in the genetic data correlates closely with geographic placement of the study sites (Figures S1 and S2, Appendix S2). In contrast, the STRUCTURE results (Figure S3, Appendix S2) indicate some substructure within the study area. The Mantel test for correlation between genetic differentiation and geographic distance indicates highly significant IBD (Figure S4, Appendix S2; *p*‐value <.00001).

### 3.2 Environmental data

We used the 13 continuous environmental variables (Table S1, Appendix S1) and carried out linear regression and PCA analyses to assess the presence of covariation and the weight of contribution of each variable. Figure S5 (Appendix S2) shows the results for the linear regression. We found strong linear relationships between NDVI, leaf area index, fPAR, evapotranspiration, and mean annual tree cover (|*R*| > .80 and *p* < .001) and (Figure S5, Appendix S2) between gross primary production and net photosynthesis (*R* value of .82 and a *p*‐value of .001). Figure S6, Appendix S2, shows the results of the PCA of environmental data, where the first and second principal components accounted for 56.6% and 18.5% of the variation among the variables, respectively. Using the results of both methods we selected a single variable (marked by asterisk in Table S1, Appendix S1) to represent all covarying factors if |*R*| was >.85. NDVI was selected to represent covarying fPAR, LAI, TC, EVI, LE, and ET based on the PCA, where NDVI explained the most variance on the first axis (56% of total variance; Figure S6, Appendix S2). PSN was selected to represent covarying GPP and elevation based on the PCA, where PSN explained the most variance on the second axis (18.5% of total variance; Figure S6, Appendix S2).

### 3.3 Pipeline outputs

#### 3.3.1 O_1_: Genetic differentiation matrix

Genetic differentiation among the 38 sites, expressed as pairwise *F*_ST_ values, is published and described in detail by Opiro et al. (2017) and is available in Table S3 (Appendix S1).

#### 3.3.2 O_2_: Circuit models of environmental connectivity

**Figure 3:**
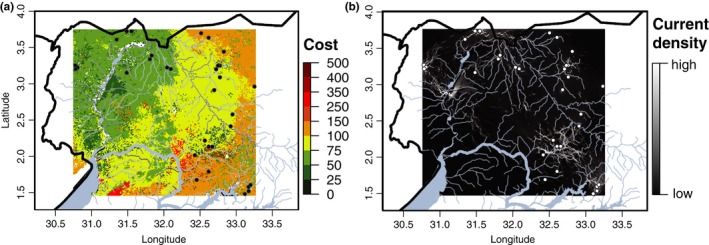
Model outputs for the top‐scoring environmental variable, net photosynthesis (PSN) obtained using Circuitscape. (a) Map showing the resistance surface costs for the only environmental variable strongly correlated with genetic differentiation, net photosynthesis (PSN, Table 1). Resistance costs vary from dark green to dark red reflecting areas of low and high resistance to tsetse movement, respectively. (b) The output of Circuitscape analysis showing the current map of the modeled connectivity expressed as current density, varying for low (black) to high (white) connectivity

Twenty resistance surfaces based on the five independent environmental variables (Figure 2: I_2_) produce 20 current maps in Circuitscape (Figure 2: O_2_). Current density (black to white scale in Figure 3b) shows the density of the modeled random‐walk dispersal pathways most likely based on the four resistance surfaces used for each of the five environmental variables. Figure 3 shows these maps for net photosynthesis because this was the variable most significantly correlated with genetic differentiation (see below). The PSN current map indicates stark contrasts between areas of low and high connectivity across northern Uganda, particularly between the Albert Nile and Lake Kyoga (Figure 3).

#### 3.3.3 O_3_: Correlation of environmental data and genetic differentiation

Results from the tests for normality and homogeneity of residuals confirmed that linearity assumptions of MMRR were not violated (Figure S7, Appendix S2). Results from the MMRR and the partial Mantel tests are presented in Table 2. Both tests indicated that PSN was the only environmental variable correlated with genetic differentiation (Table 2). There was a marginally significant negative association of *F*_ST_ with PSN when the resistance surface was scaled exponentially (Table 2; *p*‐value .050), indicating higher genetic differentiation at low PSN (high modeled connectivity at high PSN). The partial Mantel tests also detected a significant association of *F*_ST_ with low PSN (Table 2; *p*‐values .021 and .0338, respectively). None of the other four environmental variables were significant in any tests (Table 2).

#### 3.3.4 O_4_: Connectivity surface

**Figure 4:**
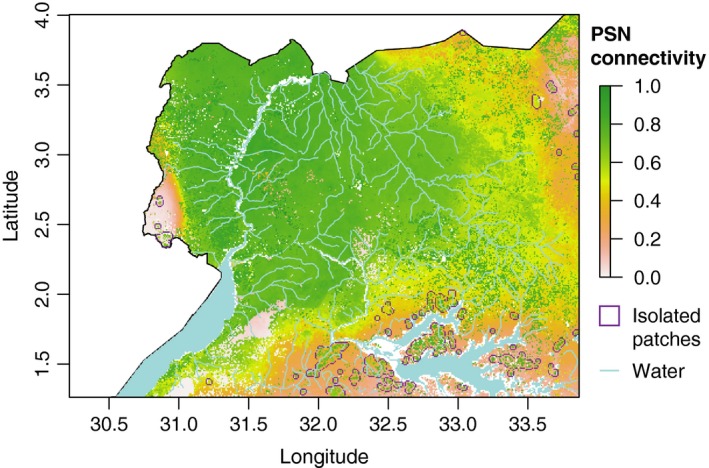
Map showing the connectivity surface based on net photosynthesis (PSN) and 317 presence data and using a univariate MaxEnt (Elith et al., 2011) analysis. The map also shows the location of discrete isolated patches in purple identified with tools implemented in R (see Figure 2 for details)

The environmental variable that was significantly correlated with genetic differentiation (PSN; Table 2) was used to create the connectivity surface. This connectivity surface indicates fine‐scale variability of expected environment‐dependent genetic differentiation in *G. f. fuscipes*, and the existence of a large region of well‐connected landscape in the northwest along the Albert Nile and the Achwa River (green in Figure 4). There is also patchy PSN on the eastern margin of the study area (pink in Figure 4), indicating low modeled connectivity among habitats in these regions.

#### 3.3.5 O_5_ and O_6_: Identifying isolated habitat patches

R tools (Figure 2: M_5_) identified one large continuous suitable habitat patch and 1351 smaller ones. The main habitat patch, representative of major *G. f. fuscipes* dispersal, extends from Uganda's western border with the Democratic Republic of the Congo to the 33° latitude line and from the shores of Lake Kyoga north to the nation's border with South Sudan (Figure 4). Using the grouping criteria defined in the methods section (a final cutoff score of >0.5, a minimum size of 4 pixels, and a clustering range of 5 km), most of the 1,351 discrete patches were eliminated from further analyses during the clustering step to avoid including patches with low probability of being able to sustain tsetse populations (too small) or high risk of re‐invasion (too close to other patches). This left 40 patches after removing patches less than 4 km^2^ in size or within 5 km of other patches.

**Figure 5:**
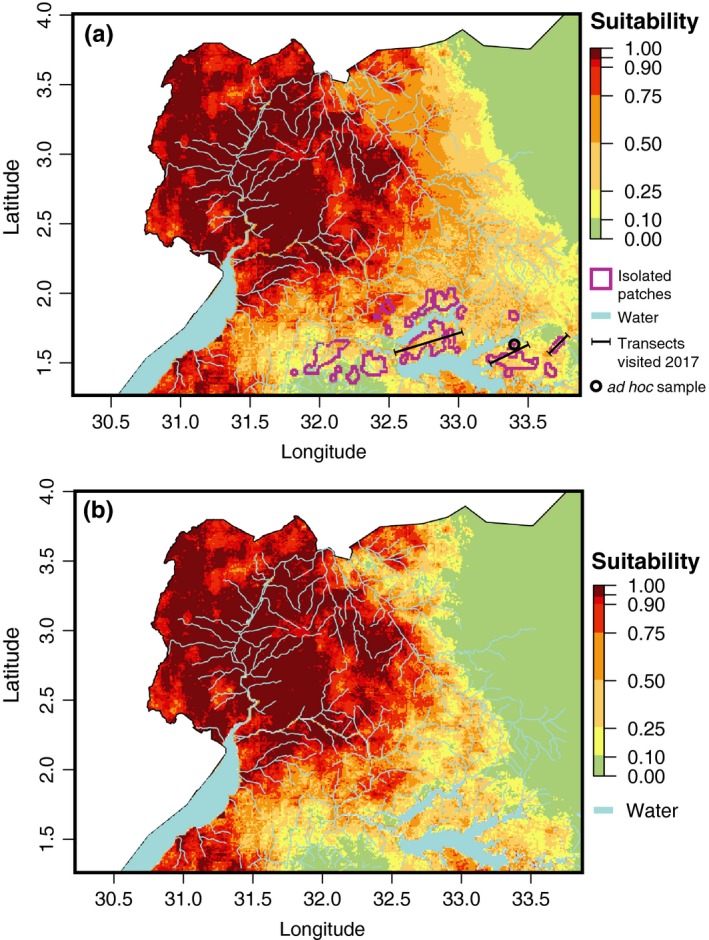
Habitat suitability maps for *G. f. fuscipes* in northern Uganda: (a) updated habitat suitability map obtained using 317 presence data, 12 environmental variable relevant to tsetse ecology (Table 1), and a canonical multivariate MaxEnt (Elith et al., 2011) analysis. This map also shows the twenty‐four isolated patches identified by the model (gray polygons), the three transects (black segments) used for the field survey, and the location of the tsetse sample from one of the isolated patches used to validate the method; (b) FAO habitat suitability map for *G. f. fuscipes* (Wint & Rogers, 2000). The legend to the right of each map explains the map color scheme, ranging from dark red (highly suitable habitat) to green (unsuitable habitat). Water bodies are shown in light blue

Figure 5a shows the updated habitat suitability map for *G. f. fuscipes* in northern Uganda obtained after merging the new MaxEnt habitat model with the existing FAO suitability model shown in Figure 5b (Wint & Rogers, 2000). The updated suitability model confirms a large continuous area of suitable G*. f. fuscipes* habitat, visualized in dark red, that covers approximately 46,500 km^2^ in the northwestern and parts of western Uganda and a patchy edge of less suitable habitat along the eastern edges of this region visualized in orange and yellow (Figure 5a). Notably, the habitat suitability model (Figure 5a) extends over a smaller area than the connectivity surface (Figure 4). This is expected because the habitat suitability model takes into consideration more variables that might represent ecological or life‐history requirements that are not correlated with genetic differentiation beyond IBD. This updated model (Figure 5a) matches quite well with the FAO model (Figure 5b) in their area of overlap, but importantly, it also extends ~60 km further to the northeast. This equates to ~20,000 km^2^ more landscape that is suitable for *G. f. fuscipes* in northeastern Uganda than previously recognized.

Of the forty discrete landscape patches of adequate size and distance (purple outlines in Figure 4), twenty‐four fell within habitat modeled at greater than 25% suitability for *G. f. fuscipes* (purple outlines in Figure 5a), so were retained as possible candidates for local eradication and/or testing of novel control methods. All these patches fall north and east of Lake Kyoga, in the southern parts of the study area.

### 3.4 Validation of the methodological pipeline using field and genetic data

**Figure 6:**
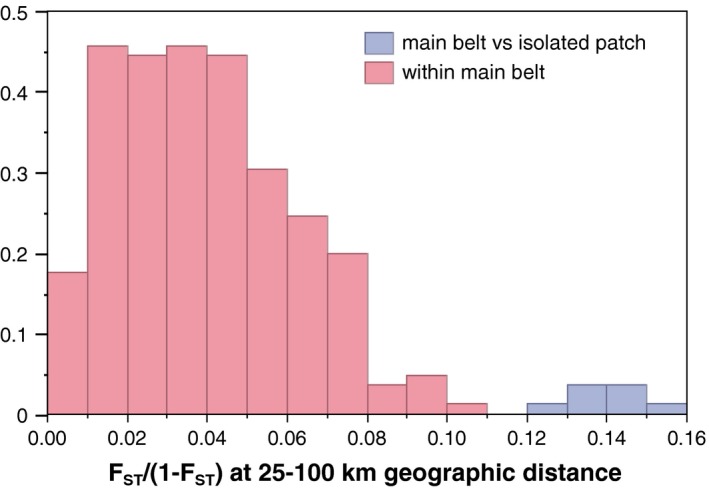
Histogram of genetic differentiation found between samples at geographic distances of 25–100 km. Pairwise estimates from within the main continuous habitat are shown in red, and pairwise estimates including the *ad hoc* sample from the model‐predicted isolated patch are shown in blue. *F*_ST_ was computed in ARLEQUIN (Excoffier & Lischer, 2010; Wright, 1951) adjusted for finite populations (Rousset, 1997) using the equation *F*_ST_/(1 − *F*_ST_)

The field survey of the transects spanning three model‐defined isolated patches of suitable habitat captured flies (18 samples) only in one location north of Lake Kyoga at Bugondo, Serere district (1.635°N, 33.290°E, Figure 5a). The absence of flies in the other locations was likely due to the fact that the survey was carried out during a very hot and dry time. In these conditions, flies tend to take refuge in vegetation thickets and thus cannot be trapped easily. Table S5 (Appendix S1) shows the *F*_ST_ pairwise estimates between flies from this isolated patch and flies from the sampling sites in main continuous habitat. Estimates of genetic differentiation [*F*_ST_ /(1 − *F*_ST_)] between samples within the main continuous habitat separated by 25–100 km averaged 0.04 (range: 0.00–0.11). In contrast, estimates from pairs of samples separated by 25–100 km that included the isolated patch averaged 0.14 (range: 0.12–0.16). This difference was statistically significant, according to a *t* test *p*‐value <.0001 (Figure 6). This result suggests that the model we built correctly identified isolated habitat patches with relative high genetic differentiation compared to pairs of samples located in more continuous habitat and that PSN, although only marginally significant, is useful to identify isolated habitat patches in this species.

## 4 DISCUSSION

### 4.1 Population differentiation and genetic diversity of G. fuscipes fuscipes in the study area

The analyses of genetic differentiation among the sampling sites used in this study (a subset of the Opiro et al., 2017 study) indicated a smooth transition of genetic variance across geographic space that closely reflects geographic distance as shown by the results of the PCA and DAPC analyses (Figures S1 and S2, Appendix S2), some substructure based on STRUCTURE results (Figure S3, Appendix S2), and strong IBD (Figure S4, Appendix S2). Although these results align with the findings of Opiro et al. (2017), with the analysis added here, our interpretation is somewhat different. Recent work has shown that strong IBD can create a false signal of population structure (Frantz et al., 2009; Meirmans et al., 2012; Falush et al., 2016) and can result in a STRUCTURE pattern that looks like a smooth transition between two genetic clusters, which is exactly what we find at *K* = 2 in this study area (Figure S3, Appendix S2). Given these considerations, we suggest that there is little evidence of genetic structure beyond IBD in the study area. This is different from the conclusion drawn by Opiro et al., 2017; where the observed zone of genetic admixture, the so‐called the transition zone, was interpreted to be the result of secondary contact between two genetically distinct clusters. This conclusion is strengthened by the fact that there is a smooth gradient of allelic richness from northwest to northeast (Table 1; Opiro et al., 2017), and by the finding of high migration rates and highly admixed individuals reported for this region that suggests free interbreeding (Opiro et al., 2017). Nonetheless, this is an important point to take into account because genetic structure caused by past allopatry, if present, can limit the accuracy of MMRR in detecting correlation of genetic differentiation and environmental variables. Here, we take this possibility into account in the interpretation of PSN as a good predictor of genetic differentiation (below) and urge any future work that includes more than a single genetic cluster to incorporate genetic structure explicitly into tests for correlation using approaches that use population‐specific covariates (Gautier, 2015).

Another important factor to consider in landscape genetics is the possible artifacts caused by demographic history. For instance, founder events can lead to genetic drift, which inflates genetic differentiation in isolated populations, and can confound landscape genetics interpretations by uncoupling patterns of differentiation from its environmental drivers. For this reason, we consider Ne estimates and bottleneck tests results (Opiro et al., 2017), which are presented in Table 1. Ne estimates varied considerably in size (average ~425; range = 17–1,549), with wide confidence intervals and some estimates not available because they were indistinguishable from infinity (Table 1; Opiro et al., 2017). Despite evidence of relatively small and variable Ne estimates, tests for bottlenecks were positive in only a few populations (sites 14, 26, 35, and 36; Figure 1, Table 1) that had been the target of recent vector control (Opiro et al., 2016, 2017). The wide range of Ne estimates and scattered evidence of bottlenecks are probably due to a combination of several factors, including limitation of the methods to estimate Ne and bottlenecks, and artifacts caused by available trapping methods. In particular, tests for bottlenecks used by Opiro et al. (2017) are not very powerful (Leblois et al., 2014), and it is possible that population fluctuations may have occurred that were not detected. Artifacts are also possible because of the fact that tsetse traps tend to favor flies that are actively searching for a blood meal at that time, and are thus likely to capture the nonrandom subset of the population that is reproductively mature and in high general condition. In addition, tsetse density in the traps tends to vary between dry and wet seasons, with much lower densities during the dry season when the majority of tsetse flies remain in vegetation thickets to avoid desiccation. A combination of these factors could explain the wide range of Ne estimates and inconclusive estimates of bottlenecks that we obtained in this and previous studies and strongly suggests the need for future studies that are specifically designed to estimate Ne and historical demographic patterns in *G. f. fuscipes*, a scope beyond the goals of this study. Nonetheless, given possible limitations and the potential impact of genetic drift in inflating differentiation during bottlenecks, we interpret correlation of environment with genetic differentiation with caution (see below).

### 4.2 Environmental‐dependent genetic differentiation

Results from the MMRR and the partial Mantel tests (Figure 2: M_3_) indicated that net photosynthesis, PSN, is the strongest predictor of tsetse fly genetic differentiation beyond that expected based on geographic distance alone (Table 2), and support the use of PSN in the next step of the pipeline, maximum entropy modeling (Figure 2: M_4_). The correlation of PSN with *F*_ST_ aligns with previous work on *G. f. fuscipes* in Uganda and our knowledge of its ecology (Dyer et al., 2011; Pollock, 1982; Rogers, 1977). The significant correlation of low PSN with genetic differentiation (Table 1) suggests that *G. f. fuscipes* tends to avoid landscape with low vegetation cover. The reason for this avoidance may be physiological or ecological, or a combination of both, and most likely reflects *G. f. fuscipes’* preference for habitat with low temperatures and high humidity (Dyer et al., 2011; Hargrove & Brady, 1992; Pollock, 1982) that often occurs near water sources along rivers and wetlands (Beadell et al., 2010; Katunguka‐Rwakishaya & Kabagambe, 1996). Habitat along rivers and wetlands would also sustain higher plant growth than drier landscape further from a water source and could account for the correlation with PSN.

Assuming that PSN is a reliable predictor of dispersal patterns and risk of *G. f. fuscipes* recolonization following an eradication campaign (caveats below), the PSN‐based circuit map (Figures 2: O_2_ and 3B) can provide insights on the spatial matrix in which these flies thrive. Under most conditions, genetic differentiation at neutral genetic markers such as those used in this study takes hundreds to thousands of generations to accumulate and thus provides insights on dispersal patterns over hundreds to thousands of generations. The PSN circuit map (Figure 3b) indicates high connectivity along the Albert Nile, the Okole River, and the northern coastline of Lake Kyoga, suggesting that waterways represent corridors for dispersal for *G. f. fuscipes* over many generations. Evidence for long‐term connectivity along waterways has practical implications because it suggests that these areas are likely at higher risk of recolonization from neighboring areas following an eradication campaign.

Interpretation of PSN as a reliable predictor of dispersal patterns and risk of *G. f. fuscipes* recolonization following an eradication campaign takes several caveats into consideration. Correlation of PSN with *F*_ST_ beyond what is associated with geographic distance could be confounded in the MMRR analysis by either (i) population structure or (i) bottlenecks, thereby making the correlation irrelevant to ongoing patterns of migration. First, genetic structure (beyond IBD) that is not accounted for can confound results because shared evolutionary history across biogeographic barriers can create a correlation that is unrelated to dispersal patterns. Although still possible, results presented here do not indicate a deep history of allopatry between subsets of the data (Figures S1 and S2, Appendix S2), and there is no clear past or present biogeographic break in the study area. Thus, we do not think that genetic structure caused the correlation of PSN with *F*_ST_ in MMRR.

Second, population bottlenecks can confound results because rapid genetic drift inflates differentiation, and if co‐occurrence of bottlenecks and patchiness in environmental conditions exist by chance, there could be a correlation unrelated to ongoing dispersal patterns. Although still possible, results from estimates of Ne and tests for bottlenecks (Opiro et al., 2017) did not provide evidence of co‐occurrence of bottlenecks and patchiness in PSN. Instead, detected population fluctuations (Table 1) are probably related to recent human intervention (Opiro et al., 2017). Furthermore, *G. f. fuscipes* in the study area has small but relatively stable Ne. This suggests that the major patterns of genetic differentiation were created under conditions of migration‐drift balance and that bottlenecks did not confound results in this study. This conclusion is also supported by previous population genetics studies that have shown evidence of ongoing gene flow among distinct populations separated by over 100 km (Abila et al., 2008; Beadell et al., 2010; Echodu et al., 2013; Hyseni et al., 2012; Opiro et al., 2017). Perhaps genetic drift contributes to high levels of genetic differentiation across small spatial scales and is counterbalanced periodically by rare long‐distance dispersal that connects populations along the habitat corridors identified in our analyses. Taken together, results from the Circuitscape analysis and MMRR suggest that PSN can be used as a predictor of genetic differentiation and that control and monitoring activities should maximize efforts along river corridors and within wetlands in order to improve both short‐ and long‐term success of *G. f. fuscipes* suppression in northern Uganda.

### 4.3 Improved habitat suitability model

Northern Uganda is a region critical for HAT control, because it is the only place in the world where the two Hat diseases are likely to merge in the near future. Yet, the most recent suitability map for the main vector of the two parasites responsible for the diseases dated back to 2000 (Wint & Rogers, 2000), before the conclusion of civil unrest that plagued the region throughout the 1990s until ~2009 (Royo, 2008; Ruaudel & Timpsen, 2011; Welburn & Odit, 2002) and made this region difficult to access. The updated habitat suitability model provided in this study (Figure 5a) built on the previous one (Figure 5b) and provides additional insights because of the addition of more than 300 presence data points (Table S2, Appendix S1). Compared to the FAO Uganda‐wide suitability maps (Figure 5b; Wint & Rogers, 2000), our updated habitat suitability model indicates the presence of about 20,000 km^2^ more suitable habitat than previously recognized at the eastern margin of *G. f. fuscipes’* range (Figure 5a). The existence of a larger suitable habitat than previously thought needs to be taken into account when planning the spatial scale of control intervention and follow‐up monitoring activities. This is because areas previously considered not suitable for tsetse persistence were deemed as low priority for control, and thus may be enabling the persistence of small tsetse populations and be sources for re‐invasion.

### 4.4 Identification of isolated patches

The PSN‐based landscape connectivity surface (Figure 4) identified one large patch of continuous suitable habitat and 1,531 discrete patches of different sizes. Most of the discrete patches were located along the northern and northwestern edge of Lake Kyoga (Figure 4, 5A). This lake runs east to west with multiple finger‐like inlets and wetland's across most of central Uganda. The extent of its wetlands and satellite lakes marks the eastern boundary of the *G. f. fuscipes* distribution and coincides with a clear genetic break between northern and southern Uganda *G. f. fuscipes* populations (Abila et al., 2008; Aksoy et al., 2013; Beadell et al., 2010, 2010). The updated habitat suitability model reflects this by identifying patchy habitat along the eastern margin of the *G. f. fuscipes* distribution (Figure 5b). The genetic analyses confirm discontinuity in the tsetse range in this area, which further supports the contribution of PSN to patterns of genetic connectivity in tsetse populations over evolutionary time scales.

We validated the existence of isolated habitat patches by conducting a field survey and genetic analyses of one population sample from one of the isolated patches. The finding of high and significant levels of genetic differentiation (Figure 6; Table S5, Appendix S1) between flies from the isolated patch and flies from sampling sites within the main continuous habitat located at similar geographic distances confirms the ability of this methodological pipeline to detect isolated patches of suitable habitat for *G. f. fuscipes*. Although we cannot exclude that genetic bottlenecks may have inflated genetic differentiation, we suggest that PSN played a prominent role in establishing genetic differentiation of the sampled isolated patch, as we identify this isolated sampling site using a PSN‐driven model. Thus, we interpret our results as an indirect validation of our method.

The finding of discrete and spatially isolated tsetse populations is also of great practical value for vector control because it provides a series of specific locations which are both ecologically and genetically isolated from the rest of the species distribution. These locations are likely to be at low risk of re‐invasion from neighboring populations from the main habitat belt providing a natural setting for experiments, akin to island settings, which have been used in the past to test new control methods such as sterile male techniques (Vreysen et al., 2000). These discrete and spatially isolated populations could be used to explore the feasibility of new control approaches, such as genetic‐based ones including transgenesis and paratransgenesis (reviewed in McGraw & O'Neill, 2013), because of their lower risk of migration in/out of the area and thereby reduced recolonization from neighboring sites outside the control area. However, as with any control measure, caution should be practiced because long‐range migration is also known to occur in this system (Beadell et al., 2010; Krafsur, Marquez, & Ouma, 2008; Opiro et al., 2017).

### 4.5 Comparison with other methods

The pipeline we describe (Figure 2) and implement in this article is not the first one to propose the use of genetic data to identify isolated tsetse populations, as it builds on advancements by Bouyer et al. (2015) and others (Biek & Real, 2010; Bouyer & Lancelot, 2017; Dicko et al., 2014; Kulkarni, Desrochers, & Kerr, 2010; Laporta, Ramos, Ribeiro, & Sallum, 2011). However, it differs from previous methods in several ways that we suggest improve our predictive power to describe tsetse movement over both ecological and evolutionary time scales.

The main difference of this pipeline from previous methods is the separate use of landscape genetics and field‐survey data, first to identify the environmental factors important in genetic differentiation over multiple generations (MMRR, Figure 2: M_1‐3_), and second to build a model of habitat isolation based on current‐day conditions and tsetse presence (Figure 2: M_4_ and M_5_). This is different than the approach used by Bouyer et al. (2015), wherein genetic differentiation between populations was used to parameterize a landscape friction map that identified genetically isolated patches in a single step. This single step approach limits the spatial observation data to the same 37 GPS points used for the genetic analysis (Bouyer et al., 2015). However, Bouyer et al.'s (2015) approach has the advantage of removing the need to assign resistance costs, a step often criticized because of its subjectivity (Manel et al., 2003, 2003; Spear, Balkenhol, Fortin, McRae, & Scribner, 2010). We minimize subjectivity in this pipeline by testing four models for each variable (Table 2; positive linear, negative linear, positive exponential and negative exponential (sensu Wang et al., 2009).

Using genetic data as an input in modeling connectivity across the landscape (Figure 2: O_4a_) allows us to build an understanding of the patterns of movement along corridors of habitat over hundreds to thousands of generations. When we combine this output (Figure 2: O_3_) with models of connectivity using field‐survey presence data, we integrate the evolutionary and ecological time scales to obtain a more complete understanding of patterns of migration over hundreds to thousands of generations and current regions of high ecological suitability. This is especially relevant in this context because long‐range migration events have been described in *G. f. fuscipes* populations, as evidenced by the finding of migrants from distances of up to ~60 km (Beadell et al., 2010; Krafsur et al., 2008; Opiro et al., 2017).

We think this approach increases the practical utility of the isolated habitat patches that we identified (Figure 2: O_6_) over what could be found using a landscape friction model using GPS coordinates from a limited population genetics sample (Bouyer et al., 2015; Dicko et al., 2014; Kulkarni et al., 2010; Laporta et al., 2011), or from field‐survey data alone as would be possible from results provided by Wint and Rogers (2000). The fact that this pipeline (1) identified a habitat patch the indeed includes tsetse flies and that (2) the fly population in this patch is significantly more genetically differentiated than others located in connected habitat and separated by similar geographic distances lends support to the ability of the method to produce accurate models of past and current movement of the target species across the landscape.

## 5 CONCLUSION AND FUTURE DIRECTIONS

Our methods pipeline builds on the progress by Bouyer et al. (2015) and others (Dicko et al., 2014; Kulkarni et al., 2010; Laporta et al., 2011) to develop an approach that produce a connectivity surface to identify isolated habitat patches that reflect both genetic connectivity and ecological connectivity at a spatial scale of interest. Our goals were threefold: to identify isolated habitat patches as candidates for local eradication, to improve the *G. f. fuscipes* habitat suitability model for a particularly high disease risk region of Uganda, and to produce a pipeline that integrates historical and current species movement patterns. We achieved this by integrating genetic data from 38 *G. f. fuscipes* sampling sites in northern Uganda (Opiro et al., 2017), high‐resolution satellite imagery, and field‐survey results. By doing so, we improved our understanding of tsetse connectivity across the landscape in this region and provide relevant information for vector control by identifying areas where tsetse population occur but are at low risk of genetic exchange with other tsetse populations. These areas can then be prioritized for trials of new control and monitoring methods and improvement of old ones. Future work will include an expansion of a similar methods pipeline to the whole country and inclusion of whole genome SNP (single nucleotide polymorphisms) data to improve resolution of patterns of habitat connectivity across the genome and the landscape, and to identify genetic associations between specific regions in the genome and environmental and physiological traits relevant to disease transmission.

## CONFLICT OF INTEREST

None declared.

## AUTHORS CONTRIBUTION

AC, SA, RE, NPS, and MB were involved in the conceptualization of this study; MB, NPS, CH, and GA participated in the formal analysis; AC, SA, RE, and EAO were involved in project administration and funding acquisition and supervised the study; MB, NPS, RO, KD, and AWD investigated the study; AC and SA contributed to the resources; NPS and MB were involved in the visualization; NPS, MB, and AC wrote the original draft of the manuscript; NPS, MB, CH, and AC participated in the writing of manuscript—review and editing. All authors read and approved the final version of the manuscript.

## DATA ACCESSIBILITY

The connectivity surface and final habitat suitability map are available in raster/ArcGIS format in the DRYAD database (doi:10.5061/dryad.2v160c9).
