# Supplementary material for: A spatial genetics approach to inform vector control of tsetse flies (Glossina fuscipes fuscipes) in Northern Uganda
Source: Ecol Evol. 2018 May 4;8(11):5336–54. doi: 10.1002/ece3.4050 (PMC6010828; doi:10.1002/ece3.4050)
Supplement: Supplementary file 1 [file ECE3-8-5336-s001.pdf]

Appendix S1. Additional tables providing details on the environmental data layers included (Fig. 2: I_2), the field survey data included (Fig. 2: I_3), the genetic samples of *Glossina fuscipes fuscipes* from northern Uganda used in the landscape genetics analysis (Fig. 2: I_1), and data on the *ad hoc* sample from a model-predicted isolated patch. Table S1 provides details on the initial 13 environmental variables included, Table S2 provides the field-survey coordinates, Table S3 provides pairwise F_{ST} values, Table S4 provides the genetic and geographic distances of the 38 landscape genetics sites used in the test for isolation by distance, and Table S5 provides pairwise F_{ST} of the *ad hoc* model-predicted isolated patch with the 38 genetic samples included in the landscape genetics analysis.

Table S1. Initial thirteen environmental variables included; the broad type of variable, description, units, pixel size, time period of data collection used, and the source including the product name or number. Variables found to be independent from one another based on linear regression analysis and PCA are marked (*). See Figures S1 and S2 for details.

Type	Description	Unit	Original Pixel Size	Period	Source
Water availability	Mean annual rainfall*	mm	8 km^2	2008 - 2011	FEWS[1]<br>Dekadal (10-day)
Water availability	Latent evaporation	W / m^2	1 km^2	2008 - 2011	NTSG[2]<br>MOD16A2 (monthly)
Temperature	Mean annual daytime land surface temperatures*	Kelvin	1 km^2	2008 - 2011	USGS[3]<br>MOD11A2 (8-day)
Temperature	Mean annual nighttime land surface temperatures*	Kelvin	1 km^2	2008 - 2011	USGS[3]<br>MOD11A2 (8-day)
Vegetative	Normalized difference vegetation index (NDVI)*	NDVI	1 km^2	2008 - 2011	USGS[3]<br>MOD13A2 (16-day)
Vegetative	Enhanced vegetation index	EVI	1 km^2	2008 - 2011	USGS[3]<br>MOD13A2 (16-day)
Vegetative	Leaf area index	m^2/m^2	0.5 km^2	2008 - 2011	USGS[3]<br>MOD15A2H (8-day)
Vegetative	Mean annual tree cover	Percent	1 km^2	2010	GLCF[4]<br>MOD44B
Photosynthesi s	Net photosynthesis*	GPP – MR	0.5 km^2	2008 - 2011	USGS[3]<br>MOD17A2H (8-day)
Photosynthesi s	Gross primary production	kg C m^2	0.5 km^2	2008 - 2011	USGS[3]<br>MOD17A2H (8-day)
Photosynthesi s	Fraction of photosynthetically active radiation (fPAR)	Percent	0.5 km^2	2008 - 2011	USGS[3]<br>MOD15A2H (8-day)
Photosynthesi s	Evapotranspiration	mm / month	1 km^2	2008 - 2011	NTSG[2]<br>MOD16A2 (monthly)
Elevation	Elevation above sea level	meters (MASL)	90 m^2 (equatorial)	N/A	CGIAR-CSI[5]<br>SRTM Digital Elevation

[1] NOAA's Famine Early Warning System (http://earlywarning.usgs.gov/fews)
[2] Numerical Terradynamic Simulation Group (http://www.ntsg.umt.edu/project/mod16)
[3] United States Geologic Survey (https://lpdaac.usgs.gov/dataset_discovery/modis/modis_products_table)
[4] Global Land Cover Facility (http://glcf.umd.edu/data/vcf)
[5] Consultative Group on International Agricultural Research Consortium for Spatial Information (srtm.csi.cgiar.org)

Table S2. Field-survey geographic coordinate number (#), latitude (Lat), and longitude (Long) of 317 *Glossina fuscipes fuscipes* presence-only data points used as MaxEnt (Elith et al., 2011) model input.

#	Lat	Long	#	Lat	Long	#	Lat	Long	#	Lat	Long	#	Lat	Long	#	Lat	Long
1	3.694	31.779	56	3.378	31.995	111	3.260	32.225	166	2.607	32.939	221	2.427	32.628	276	2.270	32.520
2	3.694	31.779	57	3.378	31.995	112	3.260	32.225	167	2.607	32.938	222	2.427	32.628	277	2.270	32.520
3	3.693	31.781	58	3.378	31.993	113	3.260	32.224	168	2.607	32.938	223	2.427	32.628	278	2.270	32.520
4	3.693	31.781	59	3.378	31.993	114	3.260	32.224	169	2.607	32.937	224	2.426	32.629	279	2.270	32.520
5	3.693	31.780	60	3.378	31.993	115	3.259	32.224	170	2.607	32.937	225	2.426	32.628	280	2.269	32.521
6	3.693	31.780	61	3.377	31.994	116	3.253	31.125	171	2.607	32.937	226	2.426	32.628	281	2.269	32.522
7	3.693	31.779	62	3.377	31.994	117	3.253	31.125	172	2.517	31.597	227	2.415	32.599	282	2.189	32.412
8	3.692	31.780	63	3.377	31.994	118	3.253	31.124	173	2.517	31.597	228	2.414	32.600	283	2.080	32.677
9	3.692	31.780	64	3.306	31.745	119	3.253	31.121	174	2.517	31.597	229	2.414	32.600	284	2.080	32.676
10	3.692	31.780	65	3.306	31.745	120	3.253	31.120	175	2.515	31.588	230	2.413	32.599	285	2.080	32.676
11	3.691	31.780	66	3.306	31.745	121	3.253	31.120	176	2.494	32.712	231	2.373	32.675	286	2.080	32.676
12	3.691	31.780	67	3.306	31.117	122	3.253	31.119	177	2.493	32.714	232	2.373	32.675	287	2.080	32.676
13	3.691	31.780	68	3.306	31.117	123	3.252	31.126	178	2.493	32.713	233	2.373	32.675	288	2.079	32.676
14	3.669	32.592	69	3.305	31.119	124	3.252	31.124	179	2.493	32.712	234	2.373	32.674	289	2.079	32.676
15	3.669	32.591	70	3.305	31.118	125	3.252	31.121	180	2.492	32.713	235	2.373	32.673	290	2.079	32.676
16	3.669	32.591	71	3.305	31.117	126	3.252	31.121	181	2.492	32.694	236	2.373	32.673	291	2.079	32.676
17	3.669	32.591	72	3.304	31.120	127	3.252	31.121	182	2.492	32.694	237	2.372	32.677	292	2.078	32.676
18	3.647	31.790	73	3.304	31.120	128	3.251	31.122	183	2.492	32.694	238	2.372	32.677	293	1.925	33.157
19	3.646	31.789	74	3.304	31.120	129	3.242	32.761	184	2.485	32.012	239	2.372	32.676	294	1.925	33.157
20	3.640	31.788	75	3.304	31.119	130	3.242	32.761	185	2.467	32.568	240	2.372	32.676	295	1.925	33.156
21	3.638	31.718	76	3.304	31.119	131	3.242	32.761	186	2.467	32.565	241	2.372	32.676	296	1.925	33.156
22	3.612	32.682	77	3.294	32.782	132	3.214	31.726	187	2.448	32.661	242	2.371	32.677	297	1.925	33.156
23	3.612	32.682	78	3.293	32.783	133	3.214	31.726	188	2.448	32.661	243	2.371	32.677	298	1.909	33.160
24	3.612	32.681	79	3.293	32.783	134	3.214	31.726	189	2.448	32.661	244	2.365	32.715	299	1.908	33.160
25	3.611	32.681	80	3.293	32.782	135	3.214	31.726	190	2.448	32.661	245	2.364	32.715	300	1.908	33.160
26	3.607	31.660	81	3.293	32.782	136	3.214	31.726	191	2.448	32.661	246	2.364	32.715	301	1.908	33.160
27	3.607	31.659	82	3.292	31.122	137	3.213	31.726	192	2.448	32.661	247	2.361	32.715	302	1.847	33.153
28	3.593	31.607	83	3.292	31.120	138	3.213	31.726	193	2.448	32.660	248	2.361	32.715	303	1.847	33.153
29	3.593	31.607	84	3.291	31.119	139	3.213	31.726	194	2.448	32.660	249	2.361	32.715	304	1.847	33.153
30	3.592	31.606	85	3.291	31.119	140	3.213	31.726	195	2.448	32.660	250	2.361	32.715	305	1.847	33.152
31	3.487	32.009	86	3.281	32.855	141	3.213	31.726	196	2.447	32.661	251	2.361	32.715	306	1.847	33.152
32	3.487	32.009	87	3.281	32.854	142	3.213	31.726	197	2.447	32.661	252	2.361	32.715	307	1.847	33.152
33	3.487	32.009	88	3.281	32.854	143	3.213	31.726	198	2.446	32.659	253	2.356	32.716	308	1.847	33.152
34	3.486	32.011	89	3.281	32.854	144	3.213	31.726	199	2.446	32.659	254	2.356	32.716	309	1.846	33.151
35	3.486	32.011	90	3.272	31.133	145	3.213	31.726	200	2.446	32.659	255	2.355	32.716	310	1.846	33.151
36	3.485	32.012	91	3.272	31.133	146	3.188	31.719	201	2.428	32.629	256	2.355	32.715	311	1.804	33.104
37	3.485	32.012	92	3.272	31.133	147	3.188	31.719	202	2.428	32.629	257	2.355	32.715	312	1.803	33.104
38	3.485	32.012	93	3.272	31.130	148	3.164	32.522	203	2.428	32.628	258	2.271	32.520	313	1.803	33.104
39	3.485	32.011	94	3.267	32.208	149	3.163	32.523	204	2.428	32.628	259	2.271	32.520	314	1.803	33.103
40	3.484	32.012	95	3.267	31.135	150	3.163	32.523	205	2.428	32.628	260	2.271	32.520	315	1.802	33.103
41	3.483	32.012	96	3.267	31.135	151	3.150	31.680	206	2.427	32.630	261	2.271	32.520	316	1.802	33.103
42	3.483	32.012	97	3.266	32.208	152	3.031	32.767	207	2.427	32.630	262	2.270	32.522	317	1.674	33.537
43	3.483	32.012	98	3.266	32.208	153	3.030	32.768	208	2.427	32.630	263	2.270	32.522			
44	3.483	32.012	99	3.266	32.208	154	3.030	32.768	209	2.427	32.630	264	2.270	32.522			
45	3.483	32.012	100	3.266	32.208	155	3.019	32.756	210	2.427	32.629	265	2.270	32.522			
46	3.483	32.012	101	3.266	31.135	156	3.019	32.755	211	2.427	32.629	266	2.270	32.521			
47	3.479	31.594	102	3.265	32.209	157	2.959	32.582	212	2.427	32.629	267	2.270	32.521			
48	3.479	31.593	103	3.265	32.209	158	2.958	32.581	213	2.427	32.629	268	2.270	32.521			
49	3.478	31.593	104	3.263	31.130	159	2.957	32.582	214	2.427	32.628	269	2.270	32.521			
50	3.441	32.854	105	3.263	31.130	160	2.840	32.965	215	2.427	32.628	270	2.270	32.521			
51	3.441	32.853	106	3.262	31.134	161	2.840	32.964	216	2.427	32.628	271	2.270	32.521			
52	3.441	32.853	107	3.262	31.134	162	2.756	32.953	217	2.427	32.628	272	2.270	32.521			
53	3.378	31.995	108	3.261	32.224	163	2.756	32.953	218	2.427	32.628	273	2.270	32.521			
54	3.378	31.995	109	3.261	32.224	164	2.738	32.950	219	2.427	32.628	274	2.270	32.521			
55	3.378	31.995	110	3.261	32.224	165	2.737	32.950	220	2.427	32.628	275	2.270	32.521			

Table S3. Pairwise F_{ST} from Opiro et al. (2017) below the diagonal as estimated in ARLEQUIN (Excoffier & Lischer, 2010) with Wright's statistics (Wright, 1951), and adjusted for finite populations (Rousset et al. 2997) using the equation $F_{ST}/(1-F_{ST})$ above the diagonal.

	1	2	3	4	5	6	7	8	9	10	11	12	13
**1**		0.012	0.015	0.017	0.058	0.028	0.047	0.016	0.037	0.049	0.024	0.038	0.031
**2**	0.012		0.000	0.004	0.044	0.032	0.034	0.035	0.034	0.041	0.003	0.027	0.033
**3**	0.015	0.000		0.004	0.052	0.033	0.058	0.053	0.044	0.060	0.012	0.043	0.050
**4**	0.017	0.004	0.004		0.035	0.026	0.047	0.055	0.026	0.052	0.015	0.031	0.036
**5**	0.055	0.042	0.049	0.034		0.073	0.074	0.071	0.050	0.063	0.043	0.029	0.042
**6**	0.027	0.031	0.032	0.025	0.068		0.021	0.037	0.009	0.055	0.032	0.042	0.019
**7**	0.045	0.033	0.055	0.045	0.069	0.021		0.030	0.019	0.073	0.025	0.043	0.027
**8**	0.016	0.034	0.050	0.052	0.066	0.036	0.029		0.032	0.056	0.056	0.058	0.033
**9**	0.036	0.033	0.042	0.025	0.048	0.009	0.019	0.031		0.047	0.032	0.032	0.033
**10**	0.047	0.039	0.057	0.049	0.059	0.052	0.068	0.053	0.045		0.033	0.019	0.036
**11**	0.023	0.003	0.012	0.015	0.041	0.031	0.024	0.053	0.031	0.032		0.011	0.014
**12**	0.037	0.026	0.041	0.030	0.028	0.040	0.041	0.055	0.031	0.019	0.011		0.009
**13**	0.030	0.032	0.048	0.035	0.040	0.019	0.026	0.032	0.032	0.035	0.014	0.009	
**14**	0.030	0.028	0.044	0.036	0.045	0.022	0.034	0.033	0.023	0.015	0.013	0.014	0.005
**15**	0.035	0.026	0.047	0.031	0.051	0.036	0.035	0.040	0.046	0.056	0.014	0.035	0.017
**16**	0.056	0.055	0.076	0.051	0.076	0.069	0.069	0.065	0.069	0.073	0.046	0.067	0.043
**17**	0.092	0.084	0.119	0.100	0.124	0.070	0.077	0.085	0.081	0.086	0.078	0.086	0.061
**18**	0.036	0.037	0.055	0.044	0.055	0.016	0.050	0.046	0.033	0.046	0.018	0.031	0.013
**19**	0.047	0.052	0.068	0.067	0.057	0.042	0.054	0.042	0.048	0.065	0.033	0.045	0.018
**20**	0.040	0.037	0.060	0.047	0.063	0.036	0.049	0.047	0.042	0.049	0.022	0.037	0.016
**21**	0.039	0.029	0.057	0.052	0.067	0.051	0.060	0.045	0.062	0.062	0.045	0.054	0.035
**22**	0.035	0.043	0.060	0.052	0.054	0.031	0.060	0.041	0.042	0.053	0.032	0.027	0.012
**23**	0.042	0.043	0.062	0.044	0.052	0.039	0.070	0.062	0.049	0.050	0.025	0.034	0.028
**24**	0.036	0.045	0.058	0.045	0.048	0.036	0.061	0.040	0.032	0.043	0.045	0.032	0.023
**25**	0.052	0.062	0.073	0.067	0.057	0.045	0.082	0.054	0.054	0.058	0.046	0.055	0.026
**26**	0.044	0.042	0.055	0.053	0.050	0.052	0.078	0.042	0.042	0.061	0.042	0.056	0.035
**27**	0.053	0.058	0.079	0.066	0.058	0.058	0.079	0.052	0.049	0.045	0.063	0.051	0.031
**28**	0.047	0.045	0.057	0.051	0.042	0.055	0.080	0.061	0.044	0.053	0.029	0.025	0.026
**29**	0.054	0.062	0.079	0.068	0.052	0.054	0.078	0.057	0.054	0.065	0.060	0.044	0.025
**30**	0.050	0.073	0.080	0.078	0.069	0.068	0.098	0.068	0.075	0.078	0.047	0.060	0.042
**31**	0.059	0.067	0.085	0.076	0.064	0.057	0.086	0.064	0.067	0.075	0.055	0.062	0.032
**32**	0.059	0.058	0.082	0.070	0.053	0.075	0.109	0.066	0.080	0.059	0.068	0.050	0.031
**33**	0.064	0.089	0.098	0.079	0.078	0.069	0.117	0.081	0.076	0.095	0.081	0.075	0.050
**34**	0.083	0.100	0.107	0.082	0.072	0.087	0.126	0.101	0.088	0.121	0.095	0.089	0.067
**35**	0.076	0.100	0.108	0.094	0.089	0.081	0.114	0.090	0.083	0.108	0.090	0.085	0.067
**36**	0.057	0.080	0.080	0.065	0.075	0.058	0.109	0.075	0.065	0.087	0.073	0.065	0.051
**37**	0.074	0.097	0.096	0.077	0.081	0.080	0.130	0.095	0.073	0.102	0.094	0.077	0.069
**38**	0.072	0.090	0.105	0.087	0.086	0.084	0.121	0.092	0.080	0.110	0.078	0.081	0.066

	14	15	16	17	18	19	20	21	22	23	24	25	26
**1**	0.031	0.036	0.059	0.101	0.037	0.049	0.042	0.041	0.036	0.044	0.037	0.055	0.046
**2**	0.029	0.027	0.058	0.092	0.038	0.055	0.038	0.030	0.045	0.045	0.047	0.066	0.044
**3**	0.046	0.049	0.082	0.135	0.058	0.073	0.064	0.060	0.064	0.066	0.062	0.079	0.058
**4**	0.037	0.032	0.054	0.111	0.046	0.072	0.049	0.055	0.055	0.046	0.047	0.072	0.056
**5**	0.047	0.054	0.082	0.142	0.058	0.060	0.067	0.072	0.057	0.055	0.050	0.060	0.053
**6**	0.022	0.037	0.074	0.075	0.016	0.044	0.037	0.054	0.032	0.041	0.037	0.047	0.055
**7**	0.035	0.036	0.074	0.083	0.053	0.057	0.052	0.064	0.064	0.075	0.065	0.089	0.085

**8**	0.034	0.042	0.070	0.093	0.048	0.044	0.049	0.047	0.043	0.066	0.042	0.057	0.044
**9**	0.024	0.048	0.074	0.088	0.034	0.050	0.044	0.066	0.044	0.052	0.033	0.057	0.044
**10**	0.015	0.059	0.079	0.094	0.048	0.070	0.052	0.066	0.056	0.053	0.045	0.062	0.065
**11**	0.013	0.014	0.048	0.085	0.018	0.034	0.022	0.047	0.033	0.026	0.047	0.048	0.044
**12**	0.014	0.036	0.072	0.094	0.032	0.047	0.038	0.057	0.028	0.035	0.033	0.058	0.059
**13**	0.005	0.017	0.045	0.065	0.013	0.018	0.016	0.036	0.012	0.029	0.024	0.027	0.036
**14**		0.044	0.072	0.063	0.021	0.017	0.029	0.047	0.012	0.033	0.028	0.029	0.036
**15**	0.042		0.014	0.067	0.019	0.042	0.021	0.036	0.037	0.035	0.048	0.053	0.048
**16**	0.067	0.014		0.053	0.050	0.072	0.046	0.075	0.074	0.046	0.064	0.068	0.055
**17**	0.059	0.063	0.050		0.054	0.083	0.054	0.083	0.070	0.079	0.095	0.101	0.093
**18**	0.021	0.019	0.048	0.051		0.010	0.003	0.033	0.003	0.012	0.020	0.014	0.022
**19**	0.017	0.040	0.067	0.077	0.010		0.014	0.040	0.000	0.028	0.019	0.009	0.017
**20**	0.028	0.021	0.044	0.051	0.003	0.014		0.036	0.009	0.022	0.031	0.027	0.027
**21**	0.045	0.035	0.070	0.077	0.032	0.038	0.035		0.052	0.058	0.054	0.065	0.066
**22**	0.012	0.036	0.069	0.065	0.003	0.000	0.009	0.049		0.016	0.014	0.014	0.013
**23**	0.032	0.034	0.044	0.073	0.012	0.027	0.022	0.055	0.016		0.024	0.016	0.029
**24**	0.027	0.046	0.060	0.087	0.020	0.019	0.030	0.051	0.014	0.023		0.005	0.008
**25**	0.028	0.050	0.064	0.092	0.014	0.009	0.026	0.061	0.014	0.016	0.005		0.004
**26**	0.035	0.046	0.052	0.085	0.022	0.017	0.026	0.062	0.013	0.028	0.008	0.004	
**27**	0.025	0.063	0.063	0.084	0.036	0.026	0.031	0.072	0.024	0.033	0.013	0.005	0.010
**28**	0.031	0.048	0.071	0.102	0.024	0.033	0.034	0.072	0.026	0.018	0.010	0.017	0.020
**29**	0.030	0.059	0.076	0.103	0.027	0.025	0.041	0.066	0.020	0.035	0.009	0.010	0.015
**30**	0.048	0.069	0.090	0.123	0.043	0.036	0.047	0.087	0.035	0.032	0.041	0.027	0.028
**31**	0.038	0.054	0.080	0.101	0.021	0.021	0.028	0.077	0.015	0.030	0.016	0.000	0.011
**32**	0.034	0.065	0.073	0.111	0.042	0.033	0.050	0.083	0.027	0.029	0.016	0.015	0.008
**33**	0.050	0.076	0.103	0.137	0.034	0.030	0.057	0.095	0.035	0.035	0.023	0.015	0.038
**34**	0.076	0.090	0.119	0.158	0.056	0.051	0.075	0.118	0.055	0.057	0.043	0.044	0.057
**35**	0.058	0.091	0.116	0.133	0.045	0.035	0.062	0.119	0.042	0.048	0.042	0.028	0.049
**36**	0.052	0.072	0.086	0.133	0.040	0.031	0.053	0.102	0.037	0.035	0.014	0.020	0.038
**37**	0.060	0.100	0.121	0.153	0.055	0.047	0.076	0.113	0.044	0.056	0.035	0.042	0.060
**38**	0.059	0.090	0.117	0.135	0.043	0.043	0.066	0.118	0.035	0.049	0.041	0.038	0.046

	**27**	**28**	**29**	**30**	**31**	**32**	**33**	**34**	**35**	**36**	**37**	**38**
**1**	0.056	0.049	0.057	0.053	0.063	0.063	0.068	0.091	0.082	0.060	0.080	0.078
**2**	0.062	0.047	0.066	0.079	0.072	0.062	0.098	0.111	0.111	0.087	0.107	0.099
**3**	0.086	0.060	0.086	0.087	0.093	0.089	0.109	0.120	0.121	0.087	0.106	0.117
**4**	0.071	0.054	0.073	0.085	0.082	0.075	0.086	0.089	0.104	0.070	0.083	0.095
**5**	0.062	0.044	0.055	0.074	0.068	0.056	0.085	0.078	0.098	0.081	0.088	0.094
**6**	0.062	0.058	0.057	0.073	0.060	0.081	0.074	0.095	0.088	0.062	0.087	0.092
**7**	0.086	0.087	0.085	0.109	0.094	0.122	0.133	0.144	0.129	0.122	0.149	0.138
**8**	0.055	0.065	0.060	0.073	0.068	0.071	0.088	0.112	0.099	0.081	0.105	0.101
**9**	0.052	0.046	0.057	0.081	0.072	0.087	0.082	0.096	0.091	0.070	0.079	0.087
**10**	0.047	0.056	0.070	0.085	0.081	0.063	0.105	0.138	0.121	0.095	0.114	0.124
**11**	0.067	0.030	0.064	0.049	0.058	0.073	0.088	0.105	0.099	0.079	0.104	0.085
**12**	0.054	0.026	0.046	0.064	0.066	0.053	0.081	0.098	0.093	0.070	0.083	0.088
**13**	0.032	0.027	0.026	0.044	0.033	0.032	0.053	0.072	0.072	0.054	0.074	0.071
**14**	0.026	0.032	0.031	0.050	0.040	0.035	0.053	0.082	0.062	0.055	0.064	0.063
**15**	0.067	0.050	0.063	0.074	0.057	0.070	0.082	0.099	0.100	0.078	0.111	0.099
**16**	0.067	0.076	0.082	0.099	0.087	0.079	0.115	0.135	0.131	0.094	0.138	0.133
**17**	0.092	0.114	0.115	0.140	0.112	0.125	0.159	0.188	0.153	0.153	0.181	0.156
**18**	0.037	0.025	0.028	0.045	0.021	0.044	0.035	0.059	0.047	0.042	0.058	0.045

**19**	0.027	0.034	0.026	0.037	0.021	0.034	0.031	0.054	0.036	0.032	0.049	0.045
**20**	0.032	0.035	0.043	0.049	0.029	0.053	0.060	0.081	0.066	0.056	0.082	0.071
**21**	0.078	0.078	0.071	0.095	0.083	0.091	0.105	0.134	0.135	0.114	0.127	0.134
**22**	0.025	0.027	0.020	0.036	0.015	0.028	0.036	0.058	0.044	0.038	0.046	0.036
**23**	0.034	0.018	0.036	0.033	0.031	0.030	0.036	0.060	0.050	0.036	0.059	0.052
**24**	0.013	0.010	0.009	0.043	0.016	0.016	0.024	0.045	0.044	0.014	0.036	0.043
**25**	0.005	0.017	0.010	0.028	0.000	0.015	0.015	0.046	0.029	0.020	0.044	0.040
**26**	0.010	0.020	0.015	0.029	0.011	0.008	0.040	0.060	0.052	0.040	0.064	0.048
**27**		0.021	0.009	0.038	0.011	0.004	0.040	0.064	0.047	0.032	0.056	0.055
**28**	0.021		0.014	0.014	0.012	0.018	0.052	0.066	0.066	0.048	0.065	0.064
**29**	0.009	0.014		0.034	0.009	0.001	0.028	0.048	0.053	0.030	0.049	0.046
**30**	0.037	0.014	0.033		0.028	0.025	0.062	0.086	0.078	0.067	0.093	0.079
**31**	0.011	0.012	0.009	0.027		0.007	0.024	0.044	0.034	0.029	0.056	0.037
**32**	0.004	0.018	0.001	0.024	0.007		0.054	0.067	0.071	0.045	0.066	0.064
**33**	0.038	0.049	0.027	0.058	0.023	0.051		0.000	0.000	0.000	0.005	0.006
**34**	0.060	0.062	0.046	0.079	0.042	0.063	0.000		0.015	0.001	0.014	0.016
**35**	0.045	0.062	0.050	0.072	0.033	0.066	0.000	0.015		0.009	0.012	0.001
**36**	0.031	0.046	0.029	0.063	0.028	0.043	0.000	0.001	0.009		0.001	0.010
**37**	0.053	0.061	0.047	0.085	0.053	0.062	0.005	0.014	0.012	0.001		0.008
**38**	0.052	0.060	0.044	0.073	0.036	0.060	0.006	0.016	0.001	0.010	0.008	

Table S4. Genetic and geographic distances used in the test for isolation by distance (IBD) with a Mantel test with 10,000 randomizations (Mantel et al., 1997; Goudet et al., 1996) implemented in the adegenet R library (Jombart et al., 2008, 2011). Genetic distances are below the diagonal and were generated using Reynold's (1983) method, and geographic distances are above the diagonal and were generated using the Java based "geographic matrix generator" v1.2.3 (Erst, downloaded November 2017).

	1	2	3	4	5	6	7	8	9	10	11	12	13	14	15	16	17	18	19
**1**		4.5	2.1	0.9	65.9	56.2	63.7	85.9	80.4	98.5	100	96.2	121	119	168	176	192	183	181
**2**	0.19		5.8	4.9	68.2	56.2	62.9	85.2	79.6	99.7	101	97.6	123	121	169	177	193	185	183
**3**	0.19	0.16		2.8	67.1	58.1	65.7	87.9	82.5	100	102	97.8	122	121	170	178	193	185	182
**4**	0.21	0.19	0.18		65.0	55.3	62.9	85.1	79.7	97.6	99.4	95.4	120	118	167	175	191	182	180
**5**	0.28	0.26	0.27	0.25		33.2	44.5	53.9	52.5	38.2	44.1	35.2	55.7	54.0	109	115	128	118	115
**6**	0.25	0.26	0.26	0.26	0.32		12.7	31.5	27.1	47.2	46.3	45.9	74.2	72.3	113	122	140	134	132
**7**	0.30	0.28	0.31	0.31	0.33	0.28		22.2	16.8	49.7	46.4	49.2	78.0	76.1	110	120	140	135	134
**8**	0.20	0.24	0.27	0.28	0.30	0.27	0.27		6.0	41.3	34.4	42.3	68.9	67.2	90.2	101	123	120	120
**9**	0.25	0.25	0.26	0.24	0.28	0.23	0.26	0.24		44.5	38.4	45.1	72.6	70.8	96.0	106	128	125	125
**10**	0.26	0.25	0.28	0.27	0.29	0.29	0.33	0.27	0.27		9.4	3.3	28.5	26.6	71.0	78.1	93.7	86.6	85.2
**11**	0.25	0.21	0.23	0.24	0.28	0.29	0.29	0.30	0.27	0.26		12.2	34.6	32.9	67.7	75.9	93.7	88.4	87.7
**12**	0.24	0.23	0.25	0.24	0.23	0.28	0.29	0.28	0.24	0.20	0.23		28.7	26.9	73.9	80.8	95.7	88.1	86.5
**13**	0.22	0.23	0.26	0.25	0.25	0.23	0.26	0.23	0.24	0.23	0.22	0.18		1.9	61.1	64.2	72.7	62.1	59.7
**14**	0.22	0.22	0.26	0.25	0.26	0.24	0.27	0.24	0.22	0.19	0.22	0.19	0.16		61.8	65.2	74.3	63.9	61.5
**15**	0.23	0.22	0.26	0.24	0.27	0.26	0.28	0.25	0.27	0.28	0.23	0.24	0.19	0.25		11.9	38.6	46.9	51.1
**16**	0.29	0.29	0.32	0.29	0.32	0.33	0.34	0.31	0.32	0.32	0.30	0.31	0.26	0.31	0.21		26.9	37.2	42.1
**17**	0.34	0.33	0.38	0.36	0.38	0.32	0.34	0.33	0.33	0.33	0.34	0.33	0.29	0.29	0.30	0.29		18.2	24.4
**18**	0.24	0.24	0.28	0.26	0.28	0.22	0.30	0.26	0.24	0.26	0.23	0.23	0.18	0.20	0.20	0.28	0.27		6.2
**19**	0.27	0.28	0.30	0.31	0.29	0.28	0.31	0.26	0.28	0.30	0.27	0.26	0.20	0.20	0.25	0.31	0.32	0.18	
**20**	0.25	0.25	0.29	0.27	0.29	0.27	0.30	0.27	0.26	0.27	0.25	0.25	0.20	0.23	0.21	0.27	0.28	0.16	0.20
**21**	0.29	0.28	0.32	0.32	0.33	0.33	0.35	0.30	0.33	0.33	0.32	0.32	0.28	0.31	0.28	0.34	0.35	0.28	0.30
**22**	0.25	0.27	0.30	0.29	0.29	0.27	0.32	0.27	0.27	0.29	0.27	0.24	0.20	0.20	0.26	0.32	0.31	0.18	0.17
**23**	0.27	0.27	0.30	0.28	0.29	0.29	0.34	0.30	0.29	0.28	0.27	0.25	0.23	0.25	0.25	0.28	0.32	0.20	0.24
**24**	0.24	0.26	0.28	0.27	0.27	0.27	0.32	0.25	0.24	0.26	0.29	0.24	0.21	0.22	0.26	0.30	0.33	0.20	0.21
**25**	0.27	0.29	0.31	0.30	0.28	0.28	0.35	0.27	0.28	0.28	0.28	0.27	0.21	0.22	0.26	0.30	0.34	0.18	0.18
**26**	0.26	0.26	0.28	0.28	0.27	0.29	0.34	0.26	0.26	0.29	0.28	0.28	0.24	0.24	0.26	0.29	0.33	0.21	0.21
**27**	0.27	0.28	0.31	0.30	0.28	0.30	0.34	0.27	0.27	0.26	0.31	0.27	0.22	0.21	0.28	0.30	0.33	0.23	0.22
**28**	0.27	0.27	0.29	0.29	0.26	0.31	0.35	0.30	0.28	0.28	0.27	0.23	0.23	0.24	0.27	0.32	0.36	0.22	0.25
**29**	0.27	0.29	0.32	0.30	0.27	0.29	0.34	0.28	0.28	0.29	0.31	0.26	0.21	0.22	0.28	0.32	0.35	0.22	0.22
**30**	0.26	0.31	0.32	0.32	0.30	0.31	0.37	0.30	0.32	0.31	0.29	0.28	0.24	0.26	0.30	0.34	0.38	0.25	0.24
**31**	0.28	0.29	0.32	0.31	0.29	0.29	0.35	0.29	0.30	0.31	0.30	0.29	0.22	0.24	0.27	0.32	0.35	0.20	0.21
**32**	0.32	0.32	0.35	0.34	0.31	0.36	0.40	0.33	0.35	0.32	0.34	0.31	0.27	0.28	0.33	0.35	0.39	0.29	0.27
**33**	0.28	0.32	0.34	0.31	0.31	0.31	0.38	0.31	0.31	0.33	0.32	0.31	0.26	0.26	0.31	0.35	0.39	0.23	0.23
**34**	0.32	0.35	0.36	0.33	0.31	0.34	0.40	0.35	0.33	0.38	0.36	0.33	0.29	0.31	0.33	0.38	0.42	0.27	0.27
**35**	0.31	0.35	0.36	0.34	0.33	0.33	0.38	0.33	0.33	0.36	0.35	0.32	0.29	0.28	0.33	0.37	0.39	0.25	0.24
**36**	0.31	0.35	0.35	0.33	0.33	0.32	0.39	0.33	0.32	0.35	0.35	0.32	0.28	0.29	0.33	0.36	0.40	0.27	0.25
**37**	0.30	0.34	0.34	0.32	0.32	0.33	0.41	0.34	0.31	0.35	0.35	0.31	0.29	0.28	0.34	0.38	0.42	0.27	0.26
**38**	0.31	0.34	0.36	0.34	0.33	0.34	0.40	0.34	0.33	0.36	0.33	0.32	0.30	0.29	0.33	0.38	0.40	0.26	0.26

	20	21	22	23	24	25	26	27	28	29	30	31	32	33	34	35	36	37	38
**1**	186	186	180	233	210	214	191	198	203	203	190	216	212	271	270	270	275	275	273
**2**	187	188	182	235	213	217	194	202	206	207	194	220	216	275	274	274	278	278	277
**3**	187	187	181	234	211	214	191	199	203	203	190	217	212	272	270	270	275	275	273
**4**	185	185	179	232	209	213	190	197	202	202	189	216	212	271	269	269	274	274	272
**5**	120	120	114	167	146	151	133	141	145	146	137	165	165	216	214	214	220	220	219

	20	21	22	23	24	25	26	27	28	29	30	31	32	33	34	35	36	37	38
**6**	136	139	137	187	172	178	164	172	176	177	170	197	198	247	245	245	251	251	251
**7**	138	141	141	189	177	184	172	181	184	185	179	206	208	255	253	253	260	259	260
**8**	123	128	130	175	168	177	170	178	181	182	179	205	209	251	249	249	256	256	257
**9**	128	133	134	180	172	180	172	180	184	184	180	207	210	254	252	252	259	259	259
**10**	89.2	91.9	91.2	140	128	136	128	136	140	140	138	165	169	210	208	208	215	215	216
**11**	91.2	95.0	96.1	143	133	142	136	144	148	148	147	173	178	217	216	216	222	222	223
**12**	90.7	93.0	91.6	141	128	136	127	135	139	139	137	163	167	209	207	207	214	214	214
**13**	64.6	65.3	62.9	113	99.3	108	103	111	114	115	115	141	147	183	181	181	188	188	189
**14**	66.4	67.2	64.8	115	101	109	104	112	116	116	116	142	148	185	183	183	190	190	191
**15**	49.1	60.3	74.8	97.8	111	124	138	145	146	147	156	177	189	206	204	204	212	212	215
**16**	39.2	51.0	67.1	86.3	102	115	132	138	139	140	151	171	183	197	195	195	203	203	207
**17**	18.4	30.5	49.6	59.4	78.7	93.3	116	121	121	122	136	153	167	174	172	172	181	181	185
**18**	2.8	13.9	31.9	55.4	64.3	78.4	97.9	103	104	105	118	136	149	160	158	158	166	166	170
**19**	6.6	9.3	25.9	55.0	59.7	73.4	91.9	97.3	98.3	98.8	112	130	143	155	153	153	161	161	165
**20**		12.4	31.2	52.6	62.5	76.6	97.2	102	103	104	117	135	148	158	156	156	164	164	168
**21**	0.29		19.5	47.7	50.6	64.5	85.1	90.1	90.8	91.3	105	122	136	146	144	144	152	152	156
**22**	0.20	0.31		54.0	38.7	50.5	66.0	71.4	72.5	73.0	85.8	104	117	132	130	130	138	138	141
**23**	0.23	0.33	0.23		45.5	58.3	95.3	96.5	94.9	95.3	116	124	141	127	126	125	134	134	139
**24**	0.23	0.31	0.21	0.23		14.9	50.0	51.1	49.8	50.1	70.9	79.6	96.7	95.5	93.7	93.6	102	102	106
**25**	0.22	0.32	0.20	0.21	0.16		39.8	39.2	37.0	37.3	59.7	65.5	83.1	81.6	79.8	79.7	88.0	87.9	91.4
**26**	0.23	0.33	0.21	0.24	0.18	0.16		8.1	12.2	12.6	21.2	39.1	51.3	82.7	81.1	81.1	87.1	87.0	87.3
**27**	0.23	0.34	0.23	0.24	0.18	0.16	0.18		4.6	4.8	20.6	32.6	46.7	74.7	73.1	73.0	79.1	79.0	79.3
**28**	0.25	0.35	0.24	0.23	0.20	0.21	0.22	0.21		0.5	23.8	31.5	47.1	70.6	69.0	68.9	75.1	75.0	75.6
**29**	0.25	0.33	0.22	0.25	0.18	0.18	0.19	0.17	0.20		23.7	31.1	46.7	70.2	68.6	68.5	74.7	74.6	75.2
**30**	0.26	0.36	0.25	0.24	0.25	0.22	0.22	0.24	0.20	0.23		27.4	32.8	81.7	80.5	80.4	84.7	84.6	83.1
**31**	0.22	0.34	0.20	0.24	0.19	0.13	0.18	0.17	0.19	0.17	0.21		19.2	57.1	56.1	56.1	59.1	59.0	56.7
**32**	0.30	0.37	0.27	0.28	0.24	0.23	0.22	0.21	0.25	0.20	0.25	0.21		69.6	68.9	68.9	69.9	69.8	65.7
**33**	0.27	0.37	0.24	0.25	0.22	0.19	0.24	0.24	0.27	0.22	0.28	0.20	0.27		1.9	1.9	6.9	6.9	13.4
**34**	0.31	0.39	0.28	0.29	0.25	0.25	0.28	0.28	0.29	0.25	0.31	0.24	0.30	0.13		0.1	8.6	8.6	14.8
**35**	0.29	0.39	0.26	0.27	0.25	0.22	0.27	0.25	0.29	0.26	0.30	0.23	0.31	0.13	0.18		8.7	8.7	14.9
**36**	0.30	0.39	0.26	0.27	0.21	0.22	0.26	0.24	0.28	0.23	0.30	0.23	0.28	0.15	0.15	0.19		0.2	7.5
**37**	0.31	0.39	0.26	0.29	0.24	0.25	0.28	0.27	0.29	0.26	0.32	0.27	0.31	0.16	0.18	0.18	0.17		7.4
**38**	0.30	0.39	0.25	0.28	0.26	0.25	0.27	0.27	0.30	0.26	0.31	0.24	0.30	0.16	0.19	0.15	0.20	0.17	

Table S5. Pairwise F_{ST} of the *ad hoc* model-predicted isolated patch with the 38 genetic samples included in the landscape genetics analysis. Pairwise F_{ST} [adjusted for finite populations using the equation $F_{ST}/(1-F_{ST})$, Rousset et al. 2997], and p-value of exact tests between tsetse flies from the isolated patch identified with our pipeline and the 38 sampling sites from the main connected habitat. Estimates were calculated in ARLEQUIN v.3.5 (Excoffier and Lischer, 2010) with Wright's statistics (Wright, 1951), following the variance method developed by Weir and Cockerham (1984) and using 10,000 permutations to obtain exact p-values between all samples.

Sample pair	Geographic distance (km)	F_{ST}	$F_{ST}/(1-F_{ST})$	p-value
Isolated patch vs 1	300.71	0.113	0.127	<0.000
Isolated patch vs 2	304.65	0.107	0.120	<0.000
Isolated patch vs 3	300.79	0.117	0.133	<0.000
Isolated patch vs 4	300.04	0.102	0.114	<0.000
Isolated patch vs 5	247.09	0.108	0.121	<0.000
Isolated patch vs 6	278.77	0.135	0.156	<0.000
Isolated patch vs 7	287.25	0.166	0.199	<0.000
Isolated patch vs 8	283.95	0.147	0.172	<0.000
Isolated patch vs 9	286.64	0.111	0.125	<0.000
Isolated patch vs 10	242.69	0.131	0.151	<0.000
Isolated patch vs 11	250.40	0.097	0.107	<0.000
Isolated patch vs 12	241.66	0.104	0.116	<0.000
Isolated patch vs 13	216.25	0.117	0.133	<0.000
Isolated patch vs 14	217.80	0.126	0.144	<0.000
Isolated patch vs 15	239.33	0.105	0.117	<0.000
Isolated patch vs 16	230.21	0.095	0.105	<0.000
Isolated patch vs 17	206.79	0.154	0.182	<0.000
Isolated patch vs 18	193.04	0.097	0.107	<0.000
Isolated patch vs 19	188.32	0.125	0.143	<0.000
Isolated patch vs 20	191.13	0.106	0.119	<0.000
Isolated patch vs 21	179.27	0.131	0.151	<0.000
Isolated patch vs 22	165.28	0.123	0.140	<0.000
Isolated patch vs 23	157.72	0.086	0.094	<0.000
Isolated patch vs 24	128.70	0.105	0.117	<0.000
Isolated patch vs 25	115.05	0.110	0.124	<0.000
Isolated patch vs 26	114.84	0.101	0.112	<0.000
Isolated patch vs 27	106.78	0.118	0.134	<0.000
Isolated patch vs 28	102.95	0.086	0.094	<0.000
Isolated patch vs 29	102.51	0.123	0.140	<0.000
Isolated patch vs 30	110.96	0.123	0.140	<0.000
Isolated patch vs 31	84.31	0.123	0.140	<0.000
Isolated patch vs 32	91.78	0.126	0.144	<0.000
Isolated patch vs 33	33.64	0.119	0.135	<0.000
Isolated patch vs 34	35.47	0.118	0.134	<0.000
Isolated patch vs 35	35.55	0.136	0.157	<0.000
Isolated patch vs 36	28.01	0.112	0.126	<0.000
Isolated patch vs 37	28.10	0.122	0.139	<0.000
Isolated patch vs 38	27.89	0.128	0.147	<0.000
