# Supplementary material for: A spatial genetics approach to inform vector control of tsetse flies (Glossina fuscipes fuscipes) in Northern Uganda
Source: Ecol Evol. 2018 May 4;8(11):5336–54. doi: 10.1002/ece3.4050 (PMC6010828; doi:10.1002/ece3.4050)
Supplement: Supplementary file 2 [file ECE3-8-5336-s002.pdf]

Appendix S2. Additional figures providing details on the 38 genetic samples of *Glossina fuscipes fuscipes* from northern Uganda used in the landscape genetics analysis (Fig. 2: I_1), assessment of the independence of the environmental data layers included (Fig. 2: I_2), and tests for linearity assumptions of matrix regression with randomization (MMRR; Fig. 2: M_3). Figure S1 shows the principle components analysis (PCA) of the genetic data, Figure S2 shows the discriminant analysis of principal components (DAPC) of the genetic data, Figure S3 shows the results of the Bayesian clustering analyses of the genetic data, Figure S4 shows results from the tests for isolation by distance (IBD), Figure S5 shows the linear regressions for each pair of environmental variables, Figure S6 shows the PCA of the environmental data, and Figure S7 shows the tests for linearity assumptions of MMRR.

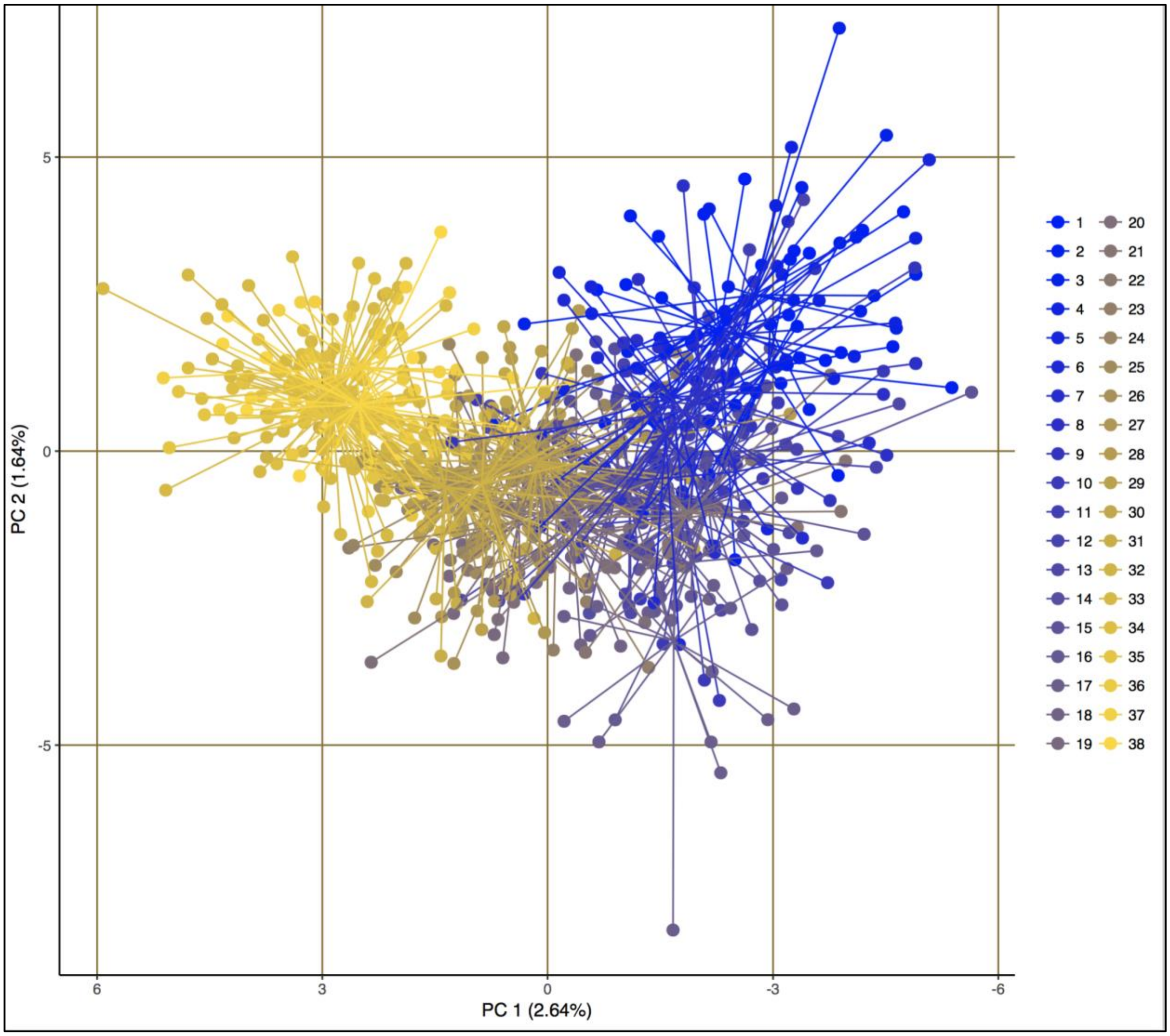

Figure S1. Principle components analysis (PCA) plots from 16 microsatellites. Points represent individual genotypes that are colored by sample, and connected by a line to the centroid of that sample according to the legend on the right, and PC 1 and 2 accounted for 2.64% and 1.64% of the variance in the microsatellite genotypes, respectively.

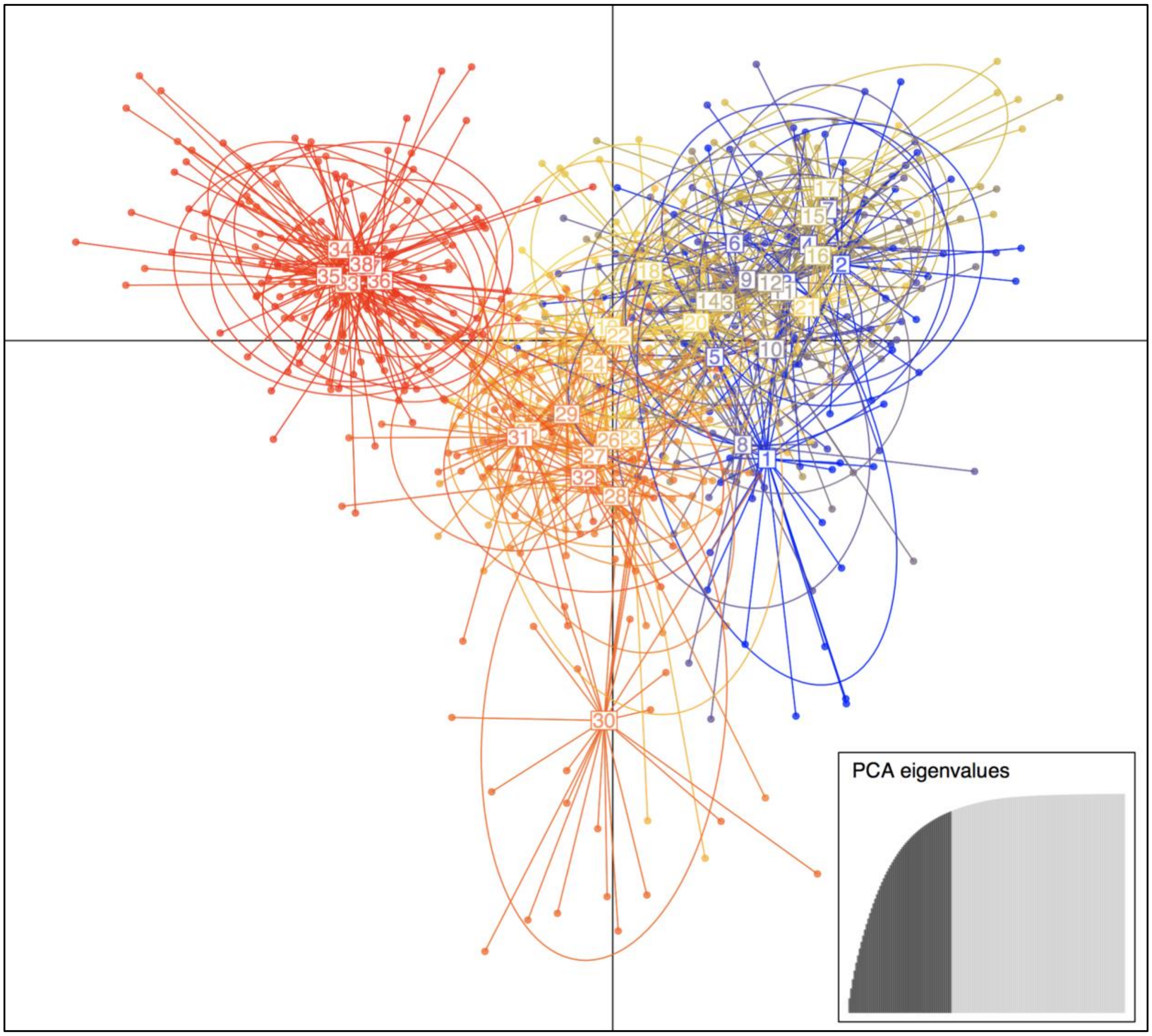

Figure S2. Results of the discriminant analysis of principal components (DAPC) using the adegenet R library (Jombart et al., 2010, 2012). Different colors represent different samples, with dots showing individuals connected by a line to the centroid and ellipse encompass 95% of the variance within each sample. The inset shows the PCA eigenvalues, with the PCs included in the discriminant function in dark shading, and based on the cross-validation formula available in the adegenet package.

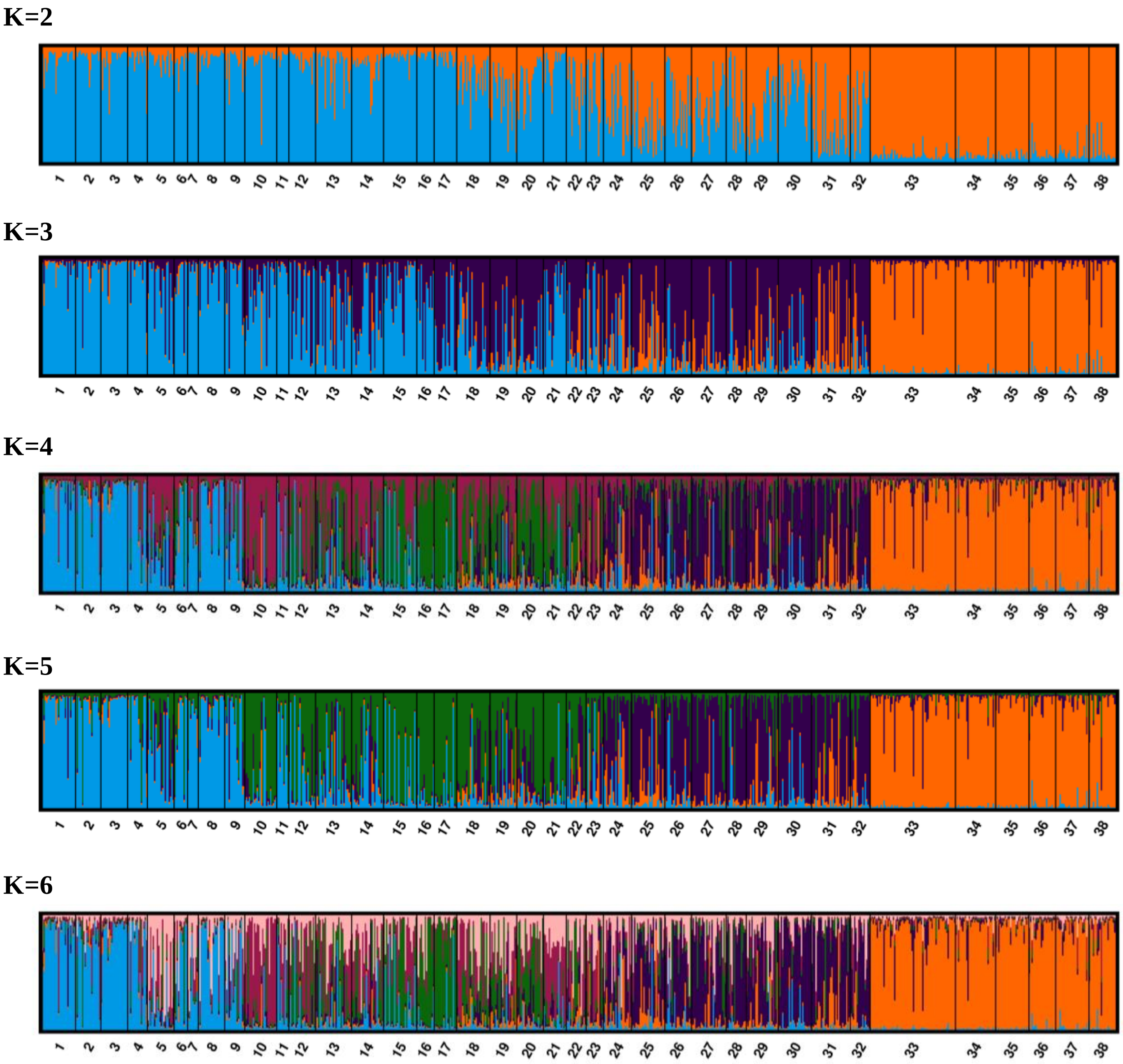

Figure S3. Plots showing the results of the Bayesian clustering analyses implemented using the program STRUCTURE v2.3.4 (Pritchard et al., 2000; Falush et al., 2003) with K=2-6. Each bar represents the composite genotype of one individual fly, the colors within the bar reflect the probability of assignment (q-value) of that individual.

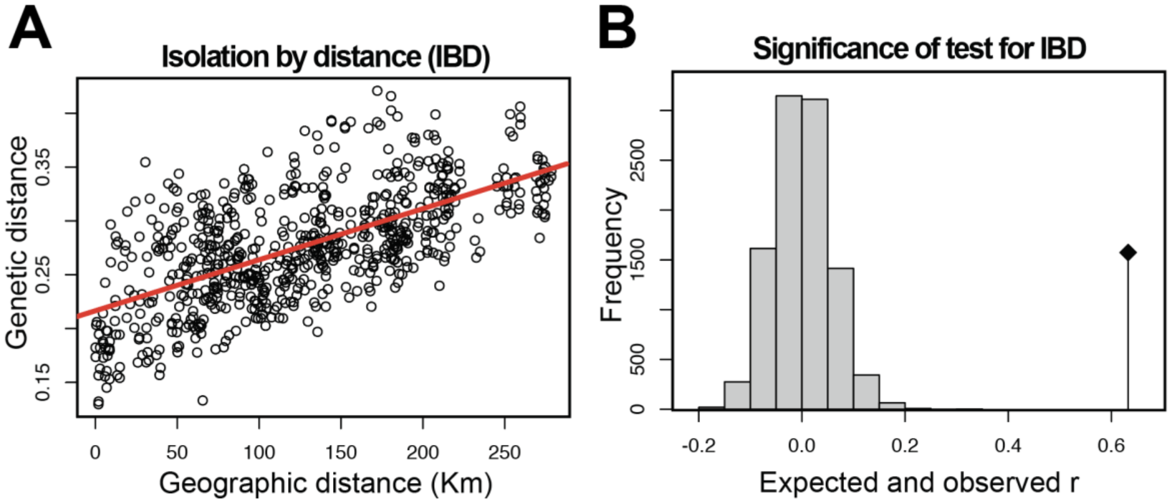

Figure S4. Results from the tests for isolation by distance (IBD) with a Mantel test implemented in the adegenet R library (Jombart et al., 2008, 2011). Panel **(A)** shows genetic distances generated using Reynold's (1983) method plotted against geographic distance in Km that were generated using the Java based "geographic matrix generator" v1.2.3 (Erst, downloaded November 2017). Panel **(B)** shows the histogram of expected value or r based on 10,000 randomizations (Mantel et al., 1997; Goudet et al., 1996), with the actual r value (0.6327), which resulted in a p-value of <0.0001, indicating highly significant levels of isolation by distance in this sample.

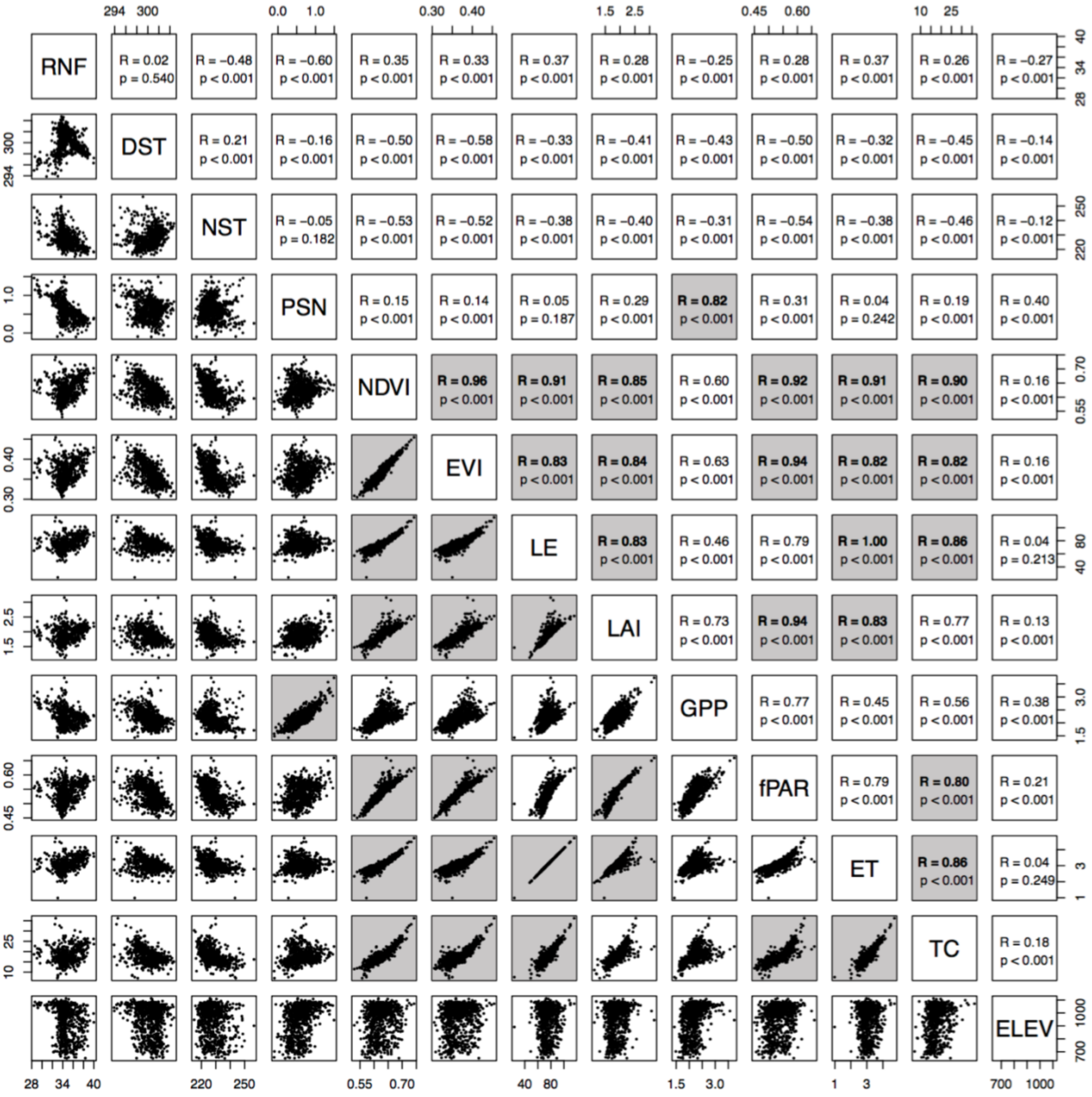

Figure S5. Linear regressions between straight path means for each pair of environmental variables: mean annual rainfall (RNF), latent evaporation (LE), mean annual daytime surface temperatures (DST), mean annual nighttime surface temperatures (NST), normalized difference vegetation index (NDVI), enhanced vegetation index (EVI), leaf area index (LAI), mean annual tree cover (TC), net photosynthesis (PSN), gross primary production (GPP), the fraction of photosynthetically active radiation (fPAR), evapotranspiration (ET), and elevation (ELEV). R and p-values pertaining to the linear regression between paired variables (listed in the diagonal) can be found above the diagonal (upper right matrix half). Visualization of the corresponding linear regressions can be found below the diagonal (lower left matrix half). Variable pairs with strong linear relationships (|R| > 0.80, p-value < 0.02) were bolded and greyed. Numbers on X and Y axes correspond to environmental variable values (see Table S1).

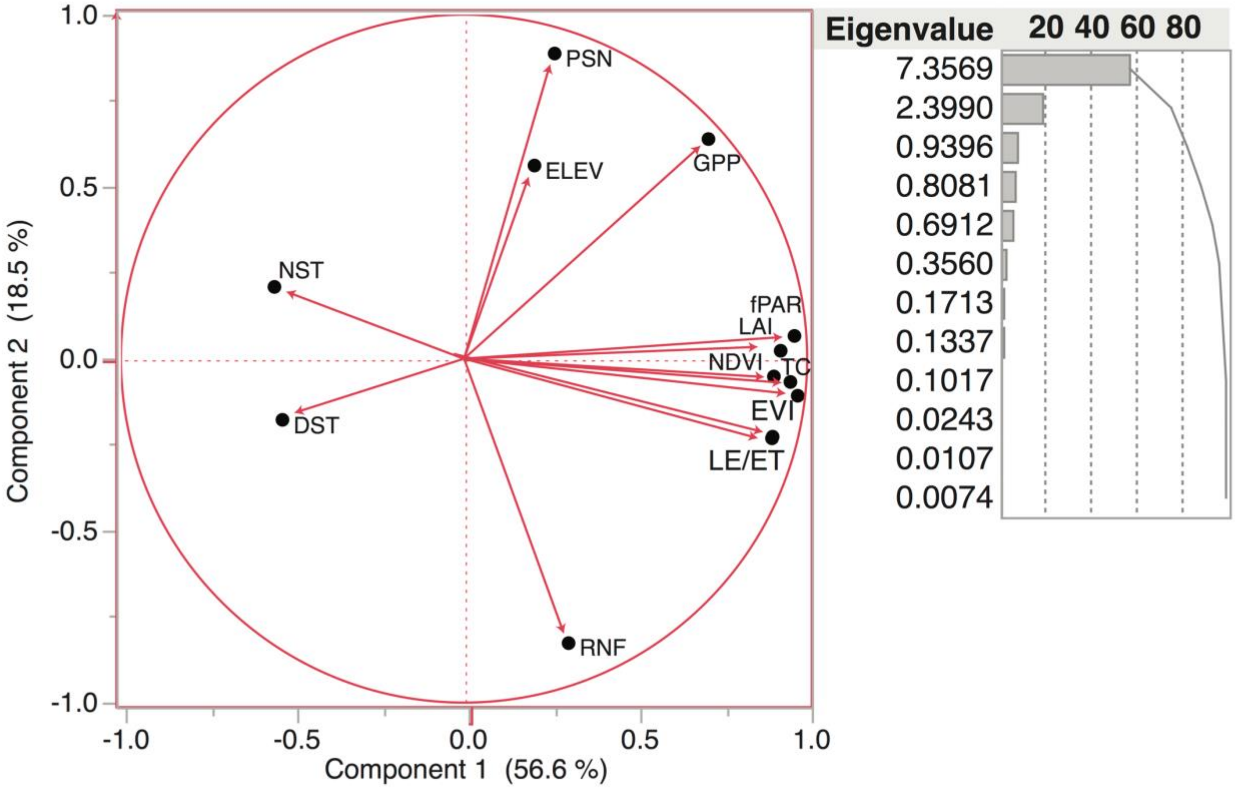

Figure S6. Results of principal components analysis of the environmental data performed in JMP v13.0 (SAS Institute Inc., Cary, NC, USA, 1989– 2016) of straight path means for each pair of variables: mean annual rainfall (RNF), latent evaporation (LE), mean annual daytime surface temperatures (DST), mean annual nighttime surface temperatures (NST), normalized difference vegetation index (NDVI), enhanced vegetation index (EVI), leaf area index (LAI), mean annual tree cover (TC), net photosynthesis (PSN), gross primary production (GPP), the fraction of photosynthetically active radiation (fPAR), evapotranspiration (ET), and elevation in meters above sea level (ELEV). The first and second principal components are represented in the graph along the horizontal and vertical axes, respectively. The graph visualizes the effect of the first two principal components (lines) on each variable (arrows). Values closer to 1 indicate greater effect of the component on the variable. The bar chart provides information on eigenvectors, representative of the variance of each principal component are provided in the bar. The variances of the twelve total components are ordered from first (top), to last (bottom).

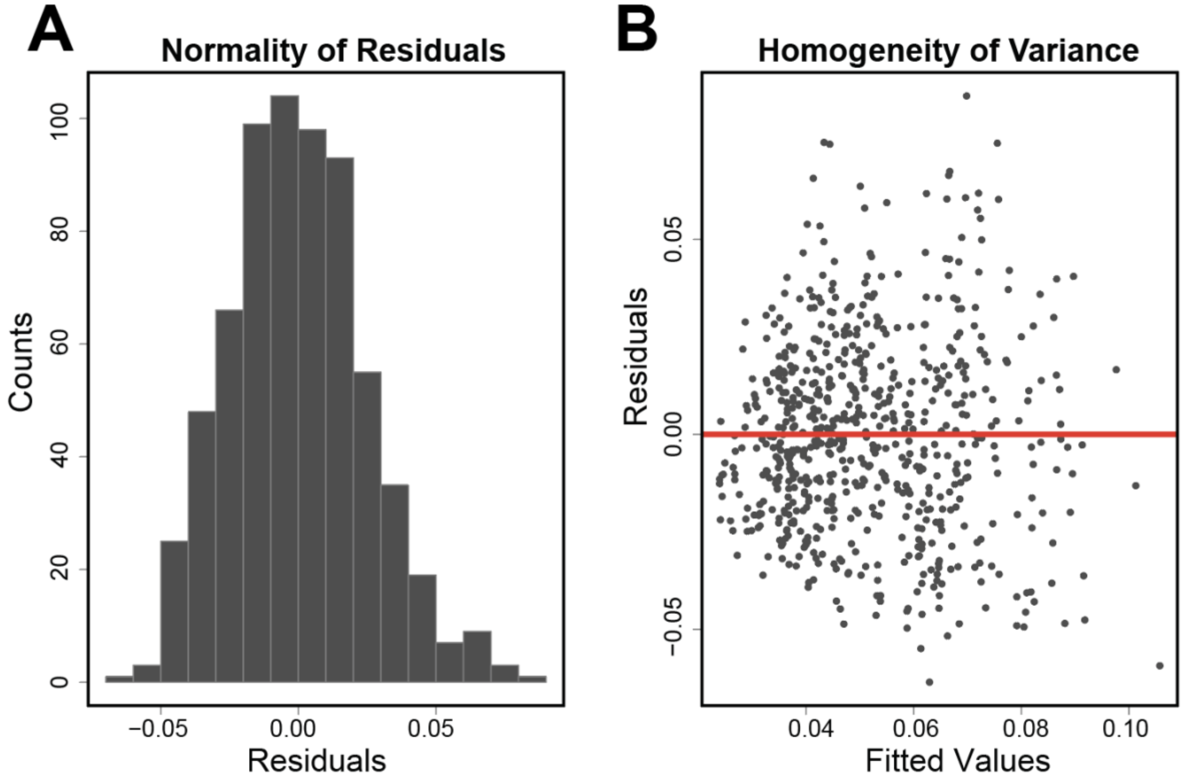

Figure S7. Tests of the linearity assumptions of multiple matrix regression with randomization (MMRR), showing F_{ST} distances on resistance distances of PSN values scaled exponentially in the negative direction. Panel **(A)** the histogram of residuals, indicating residuals are normally distributed. Panel **(B)** shows the scatter plot of the residuals versus the fitted values of the response variable (F_{ST}), indicating that the variance of the residuals is constant and does not depend on the predictor (PSN).
