# Supplementary material for: A spatial genetics approach to inform vector control of tsetse flies (Glossina fuscipes fuscipes) in Northern Uganda
Source: Ecol Evol. 2018 May 4;8(11):5336–54. doi: 10.1002/ece3.4050 (PMC6010828; doi:10.1002/ece3.4050)
Supplement: Supplementary file 3 [file ECE3-8-5336-s003.pdf]

Appendix S3. Additional methods providing details on spatial analysis in R, wherein we (i) visualize the net photosynthesis-based connectivity surface produced by maximum entropy modeling (Fig. 2: O_4; pages 1-2), (ii) identify discrete landscape patches (Fig. 2: M_5; page 3) that (iii) fall within suitable *Glossina fuscipes fuscipes* habitat (Fig. 2: M_6; page 4), and (iv) visualize alternative parameter cutoffs for identifying isolated patches displayed in Appendix S4 (pages 5-11).

```
#################################################################################################
# (i) R code to visualize output of the maximum entropy modeling (Fig. 2: O4):
#################################################################################################

#load libraries
library(raster)
library(rgdal)
library(dismo)

setwd("/Users/User/Documents/AppendixA")

scaledColor <- c("#AFCD93", "#FAF080", "#FACE7F", "#EEA32D", "#DF512A", "#D1232A", "#822024")
waterColor <- "#9AD5E6"
#################################################################################################
#load habitat HS models, prepare files for analysis:
PSN <- raster("HS-maps/PSN_raw/Glossina_fuscipes_fuscipes.asc") #maxEnt based on PSN
FAO <- raster("fuscipesf/fuscipesf/dblbnd.adf") #FAO habitat suitability model map
maxHS <- raster("HS-maps/final_maxHS/dblbnd.adf")  #max of FAO and updated habitat suitability
model
#####################################################
#load other useful shapefiles
Uganda <- shapefile("UGA_adm/UGA_adm0.dbf")
water <- shapefile("MajorWater/MajorRiversLakes.dbf")
rivers <- shapefile("MajorWater/MajorRivers.dbf")
shape <- raster("nu_hs4_aug17.txt")
values(shape)[values(shape) == 0 ] = 1
#####################################################
#crop water and turn into a Rasters (waterFull and waterCrop)
#####################################################
#create a Uganda outline and water layer for plotting:
waterFull <- crop(mask(mask(maxHS, Uganda), water, inverse=F),Uganda)
values(waterFull)[values(waterFull) > 0 ] = 0
values(waterFull)[values(waterFull) == 0 ] = 1
#crop water raster
waterCrop <- crop(waterFull,shape)
#################################################################################################

#################################################################################################
#crop each landscape surface to Northern Uganda
FAOcrop <- crop(FAOfull,shape)
#rescale to 0 to 1
r <- FAOcrop
r.min = cellStats(r, "min")
r.max = cellStats(r, "max")
r.scale <- ((r - r.min) / (r.max - r.min))
FAOcrop <- r.scale
######################
PSNcrop <- crop(PSNfull,shape)
#rescale to 0 to 1
r <- PSNcrop
r.min = cellStats(r, "min")
r.max = cellStats(r, "max")
r.scale <- ((r - r.min) / (r.max - r.min))
PSNcrop <- r.scale
######################
maxHScrop <- crop(maxHSfull,shape)
#rescale to 0 to 1
r <- maxHScrop
r.min = cellStats(r, "min")
```

```
r.max = cellStats(r, "max")
r.scale <- ((r - r.min) / (r.max - r.min))
maxHScrop <- r.scale
###############################################################################################

###############################################################################################
# Compare different maps - Northern Uganda
par(mfrow=c(3,1))
plot(FAOcrop,main="FAO habitat suitability map from 2001", col=scaledColor,
breaks=c(0,.1,.25,.5,.75,.9,.95,1))
plot(Uganda, add=T)
plot(waterFull,add=T,col=waterColor,legend=F)
plot(PSNcrop,main="PSN connectivity surface", col=scaledColor,
breaks=c(0,.1,.25,.5,.75,.9,.95,1))
plot(Uganda, add=T)
plot(waterFull,add=T,col=waterColor,legend=F)
plot(maxHScrop,main="Improved habitat suitability map", col=scaledColor,
breaks=c(0,.1,.25,.5,.75,.9,.95,1))
plot(Uganda, add=T)
plot(waterFull,add=T,col=waterColor,legend=F)
###############################################################################################

#Save the PSN connectivity surface as a pdf:
pdf(file="PSN-connectivity-surface.pdf",height=10.2,width=6.325)
# Plot habitat HS models - Northern Uganda
par(mfrow=c(2,1))
#look at the final patches on the map
plot(PSNcrop,main="PSN connectivity surface")
plot(Uganda, add=T)
plot(waterFull,add=T,col=waterColor,legend=F)
plot(rivers,add=T,col=waterColor,legend=F)

#Save the improved habitat suitability map as a pdf:
pdf(file="Improved-Habitat-Suitability.pdf",height=10.2,width=6.325)
# Plot habitat HS models - Northern Uganda
par(mfrow=c(2,1))
plot(FAOcrop,main="FAO habitat suitability map from 2001", col=scaledColor,
breaks=c(0,.1,.25,.5,.75,.9,.95,1))
plot(Uganda, add=T)
plot(waterFull,add=T,col=waterColor,legend=F)
plot(rivers,add=T,col=waterColor,legend=F)
plot(maxHScrop,main="Improved habitat suitability map", col=scaledColor,
breaks=c(0,.1,.25,.5,.75,.9,.95,1))
plot(Uganda, add=T)
plot(waterFull,add=T,col=waterColor,legend=F)
plot(rivers,add=T,col=waterColor,legend=F)
###############################################################################################
dev.off()
```

```
##################################################################################################
# (ii) R code to identify discrete landscape patches (Fig. 2: M5), after choosing cutoffs:
##################################################################################################
# HSmask3: HS > 50%, > 3 pixels
HS <- PSNcrop
HScutoff <- HS > .5
pixels <- 3
####################################################
HSfocal = focal(HScutoff, w=matrix(1,nrow=3,ncol=3), sum)
# Select patches larger than 'X out of 9' (my focus)
HSfilter3 = HSfocal > pixels
#Mask original layer with the focal pixels
HSmask3 = mask(HSfilter3, HScutoff, maskvalue=0)
values(HSmask3)[values(HSmask3) == 0 ] = NA
####################################################
#DEFINE WHICH HSmask to use, takes a long time!!!
HSmask <- HSmask3
#Buffer: put a 5 km buffer around all pixels in meters extending from both directions, so 2.5 km
+ 2.5 km = 5 km.
HSbuffer_5 = buffer(HSmask, width=2500, dissolve=T)
####################################################
#Clump these together
HSclump_5 = clump(HSbuffer_5, directions=8, gaps=F)
plot(HSclump_5)
#remove the main patch and covert all others to binary 0 or 1, 0 includes NA
patches <- HSclump_5 > 1
patches[is.na(patches)] <- 0
plot(patches)
```

```
###############################################################################################
# (iii) R code for identifying patches that fall within suitable habitat (Fig. 2: M6):
###############################################################################################
patchFocus = focal(patches, w=matrix(1,nrow=3,ncol=3), sum)
values(patchFocus)[values(patchFocus) == 0 ] = NA
values(patchFocus)[values(patchFocus) > 4 ] = NA
plot(patchFocus)
# Select pixels on edge of patches
patchOutline <- patchFocus > 1
##################################################
#look at the final patches on the map
plot(patchOutline, col="purple",legend=F, main="Patch outlines, > 50%, > 3 pixels, > 5 km apart")
plot(Uganda, add=T)

###############################################################################################
#Save Figure 4 to file:
###############################################################################################
pdf(file="PSN-connectivity-surface.pdf",height=10.2,width=6.325)
# Plot habitat HS models - Northern Uganda
par(mfrow=c(2,1))
#look at the final patches on the map
plot(PSNcrop,main="PSN connectivity surface")
plot(Uganda, add=T)
plot(waterFull,add=T,col=waterColor,legend=F)
plot(rivers,add=T,col=waterColor,legend=F)
plot(patchOutline, col="purple",legend=F, add=T)
dev.off()

###############################################################################################
#Save Figure 5A to file:
###############################################################################################
pdf(file="PSN-based-isolated-patches.pdf",height=10.2,width=6.325)
# Plot habitat HS models - Northern Uganda
par(mfrow=c(2,1))
#look at the final patches on the map
plot(patchOutline, col="purple",legend=F, main="Patch outlines, > 50%, > 3 pixels, > 5 km apart")
plot(Uganda, add=T)
plot(maxHScrop,main="Improved habitat suitability map", col=scaledColor,
breaks=c(0,.1,.25,.5,.75,.9,.95,1))
plot(Uganda, add=T)
plot(waterFull,add=T,col=waterColor,legend=F)
plot(rivers,add=T,col=waterColor,legend=F)
plot(patchOutline, col="purple",legend=F, add=T)
dev.off()
```

```
################################################################################################
# (iv) R code that produces Appendix S4 and allows for cutoff choice:
################################################################################################
#load library
library(raster)
library(rgdal)
library(dismo)

setwd("/Users/Norah/Dropbox/Caccone_Aksoy/Glossina-Mary/Mary_manuscript/Giuseppe-HS-patches")
scaledColor <- c("#AFCD93", "#FAF080", "#FACE7F", "#EEA32D", "#DF512A", "#D1232A", "#822024")
waterColor <- "#9AD5E6"
################################################################################################
#load map surfaces, prepare files for analysis:
FAO <- raster("fuscipesf/fuscipesf/dblbnd.adf") #FAO map
PSN <- raster("HS-maps/PSN_raw/Glossina_fuscipes_fuscipes.asc") #maxEnt based on PSN
maxHS <- raster("HS-maps/final_maxHS/dblbnd.adf")  #max of FAO and updated habitat suitability
model
####################################################
#load other useful shapefiles
Uganda <- shapefile("UGA_adm/UGA_adm0.dbf")
water <- shapefile("MajorWater/MajorRiversLakes.dbf")
rivers <- shapefile("MajorWater/MajorRivers.dbf")
shape <- raster("nu_hs4_aug17.txt")
values(shape)[values(shape) == 0 ] = 1
####################################################
#crop water and turn into a Rasters (waterFull and waterCrop)
####################################################
#create a Uganda outline and water layer for plotting:
waterFull <- crop(mask(mask(maxHS, Uganda), water, inverse=F),Uganda)
values(waterFull)[values(waterFull) > 0 ] = 0
values(waterFull)[values(waterFull) == 0 ] = 1
#crop water raster
waterCrop <- crop(waterFull,shape)
################################################################################################

################################################################################################
#crop each map to Northern Uganda
FAOcrop <- crop(FAOfull,shape)
#rescale to 0 to 1
r <- FAOcrop
r.min = cellStats(r, "min")
r.max = cellStats(r, "max")
r.scale <- ((r - r.min) / (r.max - r.min))
FAOcrop <- r.scale
######################
PSNcrop <- crop(PSNfull,shape)
#rescale to 0 to 1
r <- PSNcrop
r.min = cellStats(r, "min")
r.max = cellStats(r, "max")
r.scale <- ((r - r.min) / (r.max - r.min))
PSNcrop <- r.scale
######################
maxHScrop <- crop(maxHSfull,shape)
#rescale to 0 to 1
r <- maxHScrop
r.min = cellStats(r, "min")
r.max = cellStats(r, "max")
r.scale <- ((r - r.min) / (r.max - r.min))
maxHScrop <- r.scale
################################################################################################

################################################################################################
#R code to produce Appendix S4, maps 1-38: Find appropriate cutoffs of connectivity surface
scores, and minimum number of pixels
################################################################################################

SURFACE <- PSNfull
####################################################
#apply a threshold of median value
q3 <- .35
```

```
q4 <- .40
q5 <- .45
q6 <- .50
q7 <- .55
q8 <- .60
q9 <- .65
####################################################
# start PDF file for plotting maps to choose appropriate cutoffs
dev.off()
pdf(file="PSN-choose-cutoffs.pdf",height=8,width=12)
par(mfrow=c(4,6))
####################################################

SFCcutoff <- SFC > q3
###########################################################################################
SFCfocal = focal(SFCcutoff, w=matrix(1,nrow=3,ncol=3), sum)
# Select patches larger than 'X out of 9' (my focus)
SFCfilter1 = SFCfocal > 1
SFCfilter2 = SFCfocal > 2
SFCfilter3 = SFCfocal > 3
SFCfilter6 = SFCfocal > 4
SFCfilter7 = SFCfocal > 5
SFCfilter8 = SFCfocal > 6
#Mask original layer with the focal pixels
SFCmask1 = mask(SFCfilter1, SFCcutoff, maskvalue=0)
values(SFCmask1)[values(SFCmask1) == 0 ] = NA
SFCmask2 = mask(SFCfilter2, SFCcutoff, maskvalue=0)
values(SFCmask2)[values(SFCmask2) == 0 ] = NA
SFCmask3 = mask(SFCfilter3, SFCcutoff, maskvalue=0)
values(SFCmask3)[values(SFCmask3) == 0 ] = NA
SFCmask6 = mask(SFCfilter6, SFCcutoff, maskvalue=0)
values(SFCmask6)[values(SFCmask6) == 0 ] = NA
SFCmask7 = mask(SFCfilter7, SFCcutoff, maskvalue=0)
values(SFCmask7)[values(SFCmask7) == 0 ] = NA
SFCmask8 = mask(SFCfilter8, SFCcutoff, maskvalue=0)
values(SFCmask8)[values(SFCmask8) == 0 ] = NA
####################################################
#plotting maps to choose appropriate minimum SFC and density of patch
plot(SFCmask1,legend=F,main="score > 35%, > 1 pixel", col="dark green")
plot(waterCrop,add=T,col="black",legend=F)
plot(Uganda, add=T)
plot(SURFACEmask2,legend=F,main="score > .35, > 2 pixels", col="dark green")
plot(waterCrop,add=T,col="black",legend=F)
plot(Uganda, add=T)
plot(SURFACEmask3,legend=F,main="score > .35, > 3 pixels", col="dark green")
plot(waterCrop,add=T,col="black",legend=F)
plot(Uganda, add=T)
plot(SURFACEmask6,legend=F,main="score > .35, > 4 pixels", col="dark green")
plot(waterCrop,add=T,col="black",legend=F)
plot(Uganda, add=T)
plot(SURFACEmask7,legend=F,main="score > .35, > 5 pixels", col="dark green")
plot(waterCrop,add=T,col="black",legend=F)
plot(Uganda, add=T)
plot(SURFACEmask8,legend=F,main="score > .35, > 6 pixels", col="dark green")
plot(waterCrop,add=T,col="black",legend=F)
plot(Uganda, add=T)
####################################################

SURFACEcutoff <- SURFACE > q4
###########################################################################################
SURFACEfocal = focal(SURFACEcutoff, w=matrix(1,nrow=3,ncol=3), sum)
# Select patches larger than 'X out of 9' (my focus)
SURFACEfilter1 = SURFACEfocal > 1
SURFACEfilter2 = SURFACEfocal > 2
SURFACEfilter3 = SURFACEfocal > 3
SURFACEfilter6 = SURFACEfocal > 4
SURFACEfilter7 = SURFACEfocal > 5
SURFACEfilter8 = SURFACEfocal > 6
#Mask original layer with the focal pixels
SURFACEmask1 = mask(SURFACEfilter1, SURFACEcutoff, maskvalue=0)
values(SURFACEmask1)[values(SURFACEmask1) == 0 ] = NA
```

```
SURFACEmask2 = mask(SURFACEfilter2, SURFACEcutoff, maskvalue=0)
values(SURFACEmask2)[values(SURFACEmask2) == 0 ] = NA
SURFACEmask3 = mask(SURFACEfilter3, SURFACEcutoff, maskvalue=0)
values(SURFACEmask3)[values(SURFACEmask3) == 0 ] = NA
SURFACEmask6 = mask(SURFACEfilter6, SURFACEcutoff, maskvalue=0)
values(SURFACEmask6)[values(SURFACEmask6) == 0 ] = NA
SURFACEmask7 = mask(SURFACEfilter7, SURFACEcutoff, maskvalue=0)
values(SURFACEmask7)[values(SURFACEmask7) == 0 ] = NA
SURFACEmask8 = mask(SURFACEfilter8, SURFACEcutoff, maskvalue=0)
values(SURFACEmask8)[values(SURFACEmask8) == 0 ] = NA
####################################################
#plotting maps to choose appropriate minimum SURFACE and density of patch
plot(SURFACEmask1,legend=F,main="score > .4, > 1 pixel", col="dark green")
plot(waterCrop,add=T,col="black",legend=F)
plot(Uganda, add=T)
plot(SURFACEmask2,legend=F,main="score > .4, > 2 pixels", col="dark green")
plot(waterCrop,add=T,col="black",legend=F)
plot(Uganda, add=T)
plot(SURFACEmask3,legend=F,main="score > .4, > 3 pixels", col="dark green")
plot(waterCrop,add=T,col="black",legend=F)
plot(Uganda, add=T)
plot(SURFACEmask6,legend=F,main="score > .4, > 4 pixels", col="dark green")
plot(waterCrop,add=T,col="black",legend=F)
plot(Uganda, add=T)
plot(SURFACEmask7,legend=F,main="score > .4, > 5 pixels", col="dark green")
plot(waterCrop,add=T,col="black",legend=F)
plot(Uganda, add=T)
plot(SURFACEmask8,legend=F,main="score > .4, > 6 pixels", col="dark green")
plot(waterCrop,add=T,col="black",legend=F)
plot(Uganda, add=T)
####################################################

SURFACEcutoff <- SURFACE > q5
###############################################################################################
SURFACEfocal = focal(SURFACEcutoff, w=matrix(1,nrow=3,ncol=3), sum)
# Select patches larger than 'X out of 9' (my focus)
SURFACEfilter1 = SURFACEfocal > 1
SURFACEfilter2 = SURFACEfocal > 2
SURFACEfilter3 = SURFACEfocal > 3
SURFACEfilter6 = SURFACEfocal > 4
SURFACEfilter7 = SURFACEfocal > 5
SURFACEfilter8 = SURFACEfocal > 6
#Mask original layer with the focal pixels
SURFACEmask1 = mask(SURFACEfilter1, SURFACEcutoff, maskvalue=0)
values(SURFACEmask1)[values(SURFACEmask1) == 0 ] = NA
SURFACEmask2 = mask(SURFACEfilter2, SURFACEcutoff, maskvalue=0)
values(SURFACEmask2)[values(SURFACEmask2) == 0 ] = NA
SURFACEmask3 = mask(SURFACEfilter3, SURFACEcutoff, maskvalue=0)
values(SURFACEmask3)[values(SURFACEmask3) == 0 ] = NA
SURFACEmask6 = mask(SURFACEfilter6, SURFACEcutoff, maskvalue=0)
values(SURFACEmask6)[values(SURFACEmask6) == 0 ] = NA
SURFACEmask7 = mask(SURFACEfilter7, SURFACEcutoff, maskvalue=0)
values(SURFACEmask7)[values(SURFACEmask7) == 0 ] = NA
SURFACEmask8 = mask(SURFACEfilter8, SURFACEcutoff, maskvalue=0)
values(SURFACEmask8)[values(SURFACEmask8) == 0 ] = NA
####################################################
#plotting maps to choose appropriate minimum SURFACE and density of patch
plot(SURFACEmask1,legend=F,main="score > .45, > 1 pixel", col="dark green")
plot(waterCrop,add=T,col="black",legend=F)
plot(Uganda, add=T)
plot(SURFACEmask2,legend=F,main="score > .45, > 2 pixels", col="dark green")
plot(waterCrop,add=T,col="black",legend=F)
plot(Uganda, add=T)
plot(SURFACEmask3,legend=F,main="score > .45, > 3 pixels", col="dark green")
plot(waterCrop,add=T,col="black",legend=F)
plot(Uganda, add=T)
plot(SURFACEmask6,legend=F,main="score > .45, > 4 pixels", col="dark green")
plot(waterCrop,add=T,col="black",legend=F)
plot(Uganda, add=T)
plot(SURFACEmask7,legend=F,main="score > .45, > 5 pixels", col="dark green")
plot(waterCrop,add=T,col="black",legend=F)
```

```
plot(Uganda, add=T)
plot(SURFACEmask8,legend=F,main="score > .45, > 6 pixels", col="dark green")
plot(waterCrop,add=T,col="black",legend=F)
plot(Uganda, add=T)
####################################################

SURFACEcutoff <- SURFACE > q6
##################################################################################################
SURFACEfocal = focal(SURFACEcutoff, w=matrix(1,nrow=3,ncol=3), sum)
# Select patches larger than 'X out of 9' (my focus)
SURFACEfilter1 = SURFACEfocal > 1
SURFACEfilter2 = SURFACEfocal > 2
SURFACEfilter3 = SURFACEfocal > 3
SURFACEfilter6 = SURFACEfocal > 4
SURFACEfilter7 = SURFACEfocal > 5
SURFACEfilter8 = SURFACEfocal > 6
#Mask original layer with the focal pixels
SURFACEmask1 = mask(SURFACEfilter1, SURFACEcutoff, maskvalue=0)
values(SURFACEmask1)[values(SURFACEmask1) == 0 ] = NA
SURFACEmask2 = mask(SURFACEfilter2, SURFACEcutoff, maskvalue=0)
values(SURFACEmask2)[values(SURFACEmask2) == 0 ] = NA
SURFACEmask3 = mask(SURFACEfilter3, SURFACEcutoff, maskvalue=0)
values(SURFACEmask3)[values(SURFACEmask3) == 0 ] = NA
SURFACEmask6 = mask(SURFACEfilter6, SURFACEcutoff, maskvalue=0)
values(SURFACEmask6)[values(SURFACEmask6) == 0 ] = NA
SURFACEmask7 = mask(SURFACEfilter7, SURFACEcutoff, maskvalue=0)
values(SURFACEmask7)[values(SURFACEmask7) == 0 ] = NA
SURFACEmask8 = mask(SURFACEfilter8, SURFACEcutoff, maskvalue=0)
values(SURFACEmask8)[values(SURFACEmask8) == 0 ] = NA
####################################################
#plotting maps to choose appropriate minimum SURFACE and density of patch
plot(SURFACEmask1,legend=F,main="score > .5, > 1 pixel", col="dark green")
plot(waterCrop,add=T,col="black",legend=F)
plot(Uganda, add=T)
plot(SURFACEmask2,legend=F,main="score > .5, > 2 pixels", col="dark green")
plot(waterCrop,add=T,col="black",legend=F)
plot(Uganda, add=T)
plot(SURFACEmask3,legend=F,main="score > .5, > 3 pixels", col="dark green")
plot(waterCrop,add=T,col="black",legend=F)
plot(Uganda, add=T)
plot(SURFACEmask6,legend=F,main="score > .5, > 4 pixels", col="dark green")
plot(waterCrop,add=T,col="black",legend=F)
plot(Uganda, add=T)
plot(SURFACEmask7,legend=F,main="score > .5, > 5 pixels", col="dark green")
plot(waterCrop,add=T,col="black",legend=F)
plot(Uganda, add=T)
plot(SURFACEmask8,legend=F,main="score > .5, > 6 pixels", col="dark green")
plot(waterCrop,add=T,col="black",legend=F)
plot(Uganda, add=T)
####################################################

SURFACEcutoff <- SURFACE > q7
##################################################################################################
SURFACEfocal = focal(SURFACEcutoff, w=matrix(1,nrow=3,ncol=3), sum)
# Select patches larger than 'X out of 9' (my focus)
SURFACEfilter1 = SURFACEfocal > 1
SURFACEfilter2 = SURFACEfocal > 2
SURFACEfilter3 = SURFACEfocal > 3
SURFACEfilter6 = SURFACEfocal > 4
SURFACEfilter7 = SURFACEfocal > 5
SURFACEfilter8 = SURFACEfocal > 6
#Mask original layer with the focal pixels
SURFACEmask1 = mask(SURFACEfilter1, SURFACEcutoff, maskvalue=0)
values(SURFACEmask1)[values(SURFACEmask1) == 0 ] = NA
SURFACEmask2 = mask(SURFACEfilter2, SURFACEcutoff, maskvalue=0)
values(SURFACEmask2)[values(SURFACEmask2) == 0 ] = NA
SURFACEmask3 = mask(SURFACEfilter3, SURFACEcutoff, maskvalue=0)
values(SURFACEmask3)[values(SURFACEmask3) == 0 ] = NA
SURFACEmask6 = mask(SURFACEfilter6, SURFACEcutoff, maskvalue=0)
values(SURFACEmask6)[values(SURFACEmask6) == 0 ] = NA
SURFACEmask7 = mask(SURFACEfilter7, SURFACEcutoff, maskvalue=0)
```

```
values(SURFACEmask7)[values(SURFACEmask7) == 0 ] = NA
SURFACEmask8 = mask(SURFACEfilter8, SURFACEcutoff, maskvalue=0)
values(SURFACEmask8)[values(SURFACEmask8) == 0 ] = NA
####################################################
#plotting maps to choose appropriate minimum SURFACE and density of patch
plot(SURFACEmask1,legend=F,main="score > .55, > 1 pixel", col="dark green")
plot(waterCrop,add=T,col="black",legend=F)
plot(Uganda, add=T)
plot(SURFACEmask2,legend=F,main="score > .55, > 2 pixels", col="dark green")
plot(waterCrop,add=T,col="black",legend=F)
plot(Uganda, add=T)
plot(SURFACEmask3,legend=F,main="score > .55, > 3 pixels", col="dark green")
plot(waterCrop,add=T,col="black",legend=F)
plot(Uganda, add=T)
plot(SURFACEmask6,legend=F,main="score > .55, > 4 pixels", col="dark green")
plot(waterCrop,add=T,col="black",legend=F)
plot(Uganda, add=T)
plot(SURFACEmask7,legend=F,main="score > .55, > 5 pixels", col="dark green")
plot(waterCrop,add=T,col="black",legend=F)
plot(Uganda, add=T)
plot(SURFACEmask8,legend=F,main="score > .55, > 6 pixels", col="dark green")
plot(waterCrop,add=T,col="black",legend=F)
plot(Uganda, add=T)
################################################################################################

SURFACEcutoff <- SURFACE > q8
####################################################
SURFACEfocal = focal(SURFACEcutoff, w=matrix(1,nrow=3,ncol=3), sum)
# Select patches larger than 'X out of 9' (my focus)
SURFACEfilter1 = SURFACEfocal > 1
SURFACEfilter2 = SURFACEfocal > 2
SURFACEfilter3 = SURFACEfocal > 3
SURFACEfilter6 = SURFACEfocal > 4
SURFACEfilter7 = SURFACEfocal > 5
SURFACEfilter8 = SURFACEfocal > 6
#Mask original layer with the focal pixels
SURFACEmask1 = mask(SURFACEfilter1, SURFACEcutoff, maskvalue=0)
values(SURFACEmask1)[values(SURFACEmask1) == 0 ] = NA
SURFACEmask2 = mask(SURFACEfilter2, SURFACEcutoff, maskvalue=0)
values(SURFACEmask2)[values(SURFACEmask2) == 0 ] = NA
SURFACEmask3 = mask(SURFACEfilter3, SURFACEcutoff, maskvalue=0)
values(SURFACEmask3)[values(SURFACEmask3) == 0 ] = NA
SURFACEmask6 = mask(SURFACEfilter6, SURFACEcutoff, maskvalue=0)
values(SURFACEmask6)[values(SURFACEmask6) == 0 ] = NA
SURFACEmask7 = mask(SURFACEfilter7, SURFACEcutoff, maskvalue=0)
values(SURFACEmask7)[values(SURFACEmask7) == 0 ] = NA
SURFACEmask8 = mask(SURFACEfilter8, SURFACEcutoff, maskvalue=0)
values(SURFACEmask8)[values(SURFACEmask8) == 0 ] = NA
####################################################
#plotting maps to choose appropriate minimum SURFACE and density of patch
plot(SURFACEmask1,legend=F,main="score > .6, > 1 pixel", col="dark green")
plot(waterCrop,add=T,col="black",legend=F)
plot(Uganda, add=T)
plot(SURFACEmask2,legend=F,main="score > .6, > 2 pixels", col="dark green")
plot(waterCrop,add=T,col="black",legend=F)
plot(Uganda, add=T)
plot(SURFACEmask3,legend=F,main="score > .6, > 3 pixels", col="dark green")
plot(waterCrop,add=T,col="black",legend=F)
plot(Uganda, add=T)
plot(SURFACEmask6,legend=F,main="score > .6, > 4 pixels", col="dark green")
plot(waterCrop,add=T,col="black",legend=F)
plot(Uganda, add=T)
plot(SURFACEmask7,legend=F,main="score > .6, > 5 pixels", col="dark green")
plot(waterCrop,add=T,col="black",legend=F)
plot(Uganda, add=T)
plot(SURFACEmask8,legend=F,main="score > .6, > 6 pixels", col="dark green")
plot(waterCrop,add=T,col="black",legend=F)
plot(Uganda, add=T)
################################################################################################

SURFACEcutoff <- SURFACE > q9
```

```
####################################################
SURFACEfocal = focal(SURFACEcutoff, w=matrix(1,nrow=3,ncol=3), sum)
# Select patches larger than 'X out of 9' (my focus)
SURFACEfilter1 = SURFACEfocal > 1
SURFACEfilter2 = SURFACEfocal > 2
SURFACEfilter3 = SURFACEfocal > 3
SURFACEfilter6 = SURFACEfocal > 4
SURFACEfilter7 = SURFACEfocal > 5
SURFACEfilter8 = SURFACEfocal > 6
#Mask original layer with the focal pixels
SURFACEmask1 = mask(SURFACEfilter1, SURFACEcutoff, maskvalue=0)
values(SURFACEmask1)[values(SURFACEmask1) == 0 ] = NA
SURFACEmask2 = mask(SURFACEfilter2, SURFACEcutoff, maskvalue=0)
values(SURFACEmask2)[values(SURFACEmask2) == 0 ] = NA
SURFACEmask3 = mask(SURFACEfilter3, SURFACEcutoff, maskvalue=0)
values(SURFACEmask3)[values(SURFACEmask3) == 0 ] = NA
SURFACEmask6 = mask(SURFACEfilter6, SURFACEcutoff, maskvalue=0)
values(SURFACEmask6)[values(SURFACEmask6) == 0 ] = NA
SURFACEmask7 = mask(SURFACEfilter7, SURFACEcutoff, maskvalue=0)
values(SURFACEmask7)[values(SURFACEmask7) == 0 ] = NA
SURFACEmask8 = mask(SURFACEfilter8, SURFACEcutoff, maskvalue=0)
values(SURFACEmask8)[values(SURFACEmask8) == 0 ] = NA
####################################################
#plotting maps to choose appropriate minimum SURFACE and density of patch
plot(SURFACEmask1,legend=F,main="score > .65, > 1 pixel", col="dark green")
plot(waterCrop,add=T,col="black",legend=F)
plot(Uganda, add=T)
plot(SURFACEmask2,legend=F,main="score > .65, > 2 pixels", col="dark green")
plot(waterCrop,add=T,col="black",legend=F)
plot(Uganda, add=T)
plot(SURFACEmask3,legend=F,main="score > .65, > 3 pixels", col="dark green")
plot(waterCrop,add=T,col="black",legend=F)
plot(Uganda, add=T)
plot(SURFACEmask6,legend=F,main="score > .65, > 4 pixels", col="dark green")
plot(waterCrop,add=T,col="black",legend=F)
plot(Uganda, add=T)
plot(SURFACEmask7,legend=F,main="score > .65, > 5 pixels", col="dark green")
plot(waterCrop,add=T,col="black",legend=F)
plot(Uganda, add=T)
plot(SURFACEmask8,legend=F,main="score > .65, > 6 pixels", col="dark green")
plot(waterCrop,add=T,col="black",legend=F)
plot(Uganda, add=T)
#################################################################################################
dev.off()
```

```
#################################################################################################
#R code to produce Appendix S4, maps 39-42:  choose distance separating patches
#################################################################################################
## Cutoffs chosen in this case: connectivity score > .5, > 3 pixels
#start PDF file for plotting patches
dev.off()
pdf(file="PSN-patches-50percent-min3pixels-5km-to-8km-apart.pdf",height=11,width=8.5)
par(mfrow=c(3,2))
####################################################
SURFACEcutoff <- SURFACE > .5
pixels <- 3
####################################################
SURFACEfocal = focal(SURFACEcutoff, w=matrix(1,nrow=3,ncol=3), sum)
# Select patches larger than 'X out of 9' (my focus)
SURFACEfilter3 = SURFACEfocal > pixels
#Mask original layer with the focal pixels
SURFACEmask3 = mask(SURFACEfilter3, SURFACEcutoff, maskvalue=0)
values(SURFACEmask3)[values(SURFACEmask3) == 0 ] = NA
####################################################
#DEFINE WHICH SURFACEmask to use:
```

```
SURFACEmask <- SURFACEmask3
#Buffer: put a 5 km to 10 km buffer around all pixels
SURFACEbuffer_5 = buffer(SURFACEmask, width=2500, dissolve=T) #5km buffer
SURFACEclump_5 = clump(SURFACEbuffer_5, directions=8, gaps=F)
SURFACEbuffer_6 = buffer(SURFACEmask, width=3000, dissolve=T) #6km buffer
SURFACEclump_6 = clump(SURFACEbuffer_6, directions=8, gaps=F)
SURFACEbuffer_7 = buffer(SURFACEmask, width=3500, dissolve=T) #7km buffer
SURFACEclump_7 = clump(SURFACEbuffer_7, directions=8, gaps=F)
SURFACEbuffer_8 = buffer(SURFACEmask, width=4000, dissolve=T) #8km buffer
SURFACEclump_8 = clump(SURFACEbuffer_8, directions=8, gaps=F)
####################################################
#plotting map
plot(SURFACEclump_5, main="score > .5, > 3 pixels, > 5 km apart")
plot(Uganda, add=T)
plot(waterFull,add=T,col="black",legend=F)
plot(SURFACEclump_6, main="score > .5, > 3 pixels, > 6 km apart")
plot(Uganda, add=T)
plot(waterFull,add=T,col="black",legend=F)
plot(SURFACEclump_7, main="score > .5, > 3 pixels, > 7 km apart")
plot(Uganda, add=T)
plot(waterFull,add=T,col="black",legend=F)
plot(SURFACEclump_8, main="score > .5, > 3 pixels, > 8 km apart")
plot(Uganda, add=T)
plot(waterFull,add=T,col="black",legend=F)
#################################################################################################
dev.off()
```
