# Supplementary material for: A spatial genetics approach to inform vector control of tsetse flies (Glossina fuscipes fuscipes) in Northern Uganda
Source: Ecol Evol. 2018 May 4;8(11):5336–54. doi: 10.1002/ece3.4050 (PMC6010828; doi:10.1002/ece3.4050)
Supplement: Supplementary file 4 [file ECE3-8-5336-s004.pdf]

Appendix S4. Additional data providing visualization of net photosynthesis-based connectivity surface output (Fig. 2: O_4) with different parameter cutoffs for the final clumping parameters used to identify discrete landscape patches (Fig. 2: M_5). Maps 1-38 (page 1) show data used to choose the appropriate connectivity surface score cutoff (0.35-0.65) and minimum number of pixels considered (1-6). Maps 39-42 (page 2) show data used to choose the appropriate distance between connectivity surface patches (5-8 km) required to consider it "isolated".

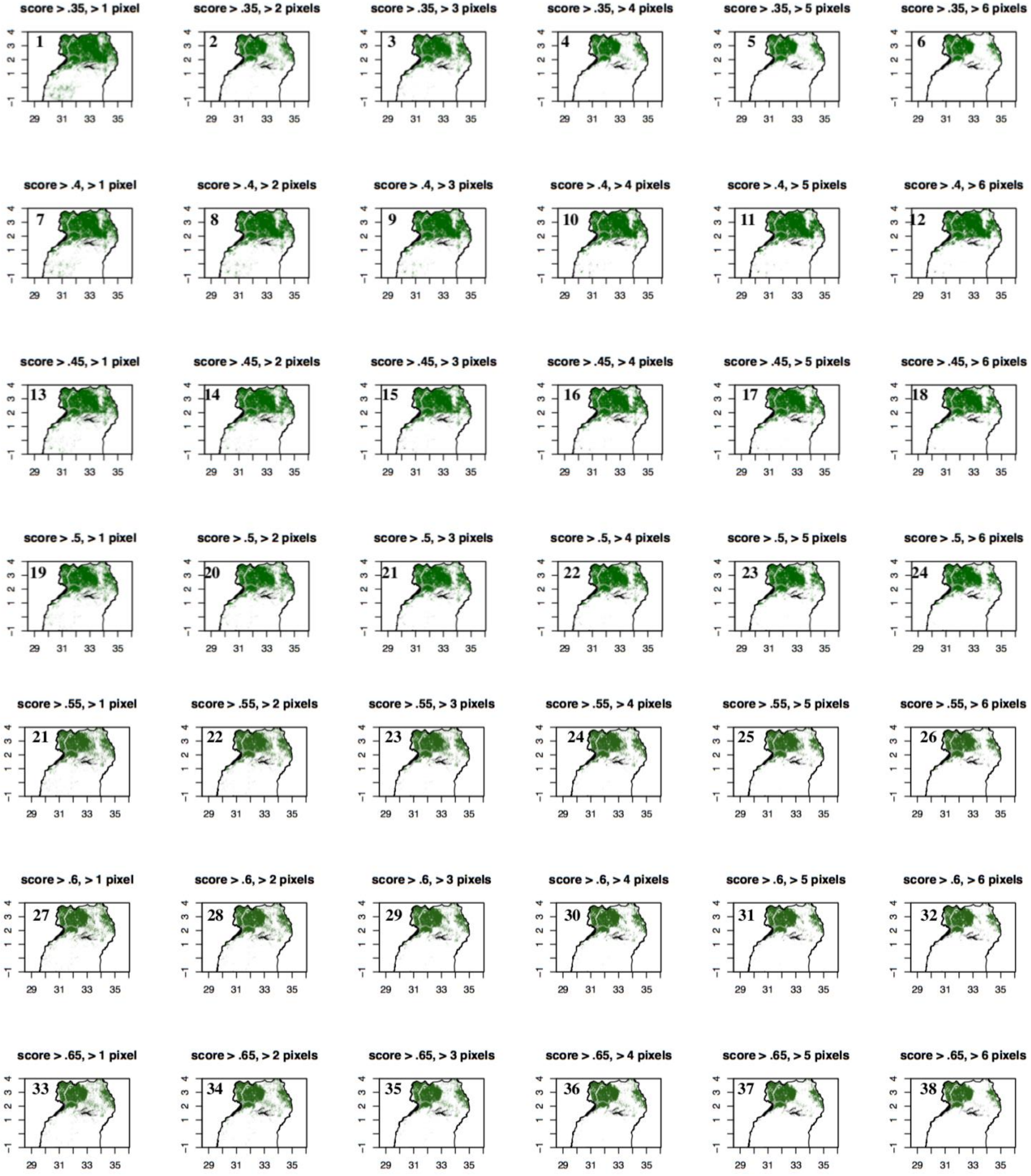

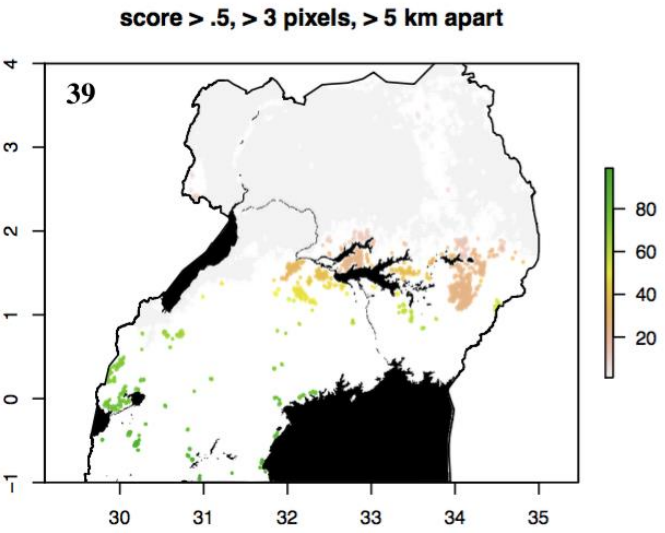
score > .5, > 3 pixels, > 5 km apart
39
4
3
2
1
0
−1
30
31
32
33
34
35
80
60
40
20

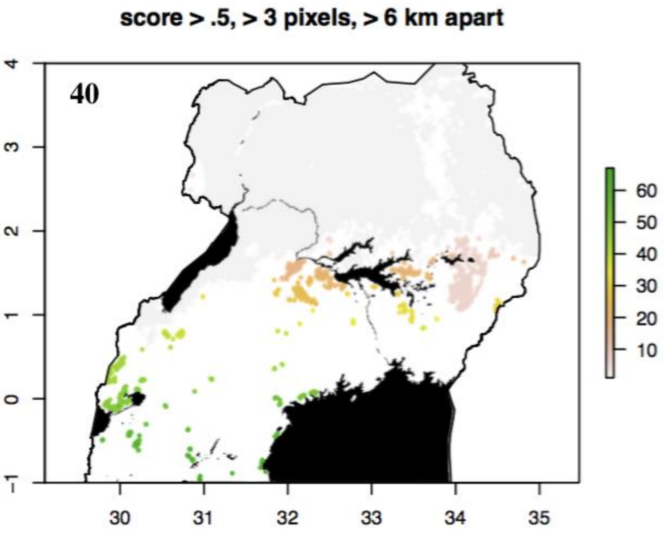
score > .5, > 3 pixels, > 6 km apart
40
4
3
2
1
0
−1
30
31
32
33
34
35
60
50
40
30
20
10

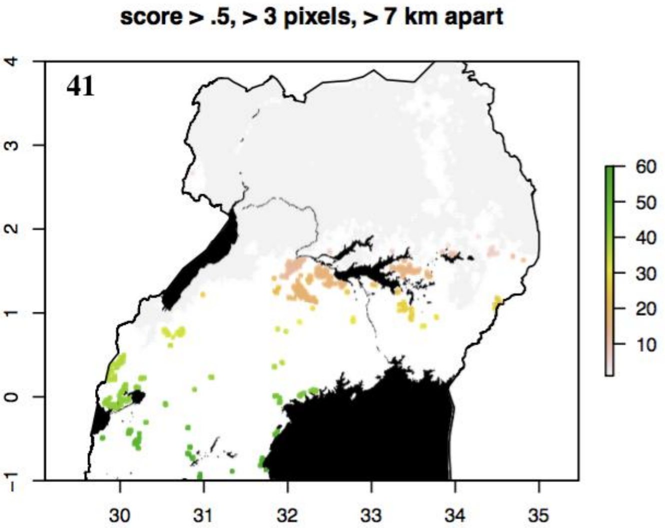
score > .5, > 3 pixels, > 7 km apart
41
4
3
2
1
0
−1
30
31
32
33
34
35
60
50
40
30
20
10

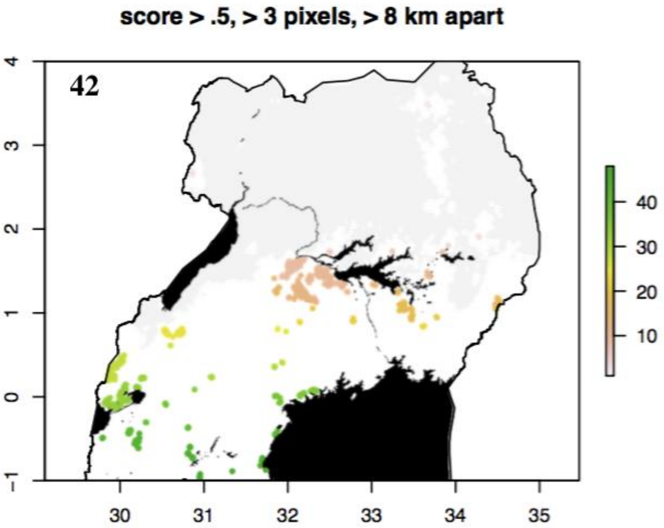
score > .5, > 3 pixels, > 8 km apart
42
4
3
2
1
0
−1
30
31
32
33
34
35
40
30
20
10
